# The effect of salidroside, an active component of *Rhodiola rosea*, on the metabolic activity of rat and human cytochromes P450 in preclinical studies

**DOI:** 10.1007/s43440-026-00842-w

**Published:** 2026-03-11

**Authors:** Eva Klaskova, Jan Jurica, Przemysław Jan Danek, Natalie Mlcuchova, Saltuk Mustafa Eyrilmez, Petra Borilova Linhartova, David Bednar, Rajamanikkam Kamaraj, Władysława Anna Daniel, Ondrej Zendulka

**Affiliations:** 1 Department of Pharmacology, Faculty of Medicine, Masaryk University, Kamenice 5, Brno, 625 00 Czech Republic; 2 Department of Pharmacology and Toxicology, Faculty of Pharmacy, Masaryk University, Palackeho trida 1, Brno, Czech Republic; 3 Masaryk Memorial Cancer Institute, Zluty kopec 7, Brno, Czech Republic; 4 Department of Pharmacokinetics and Drug Metabolism, Maj Institute of Pharmacology, Polish Academy of Sciences, Smętna 12, Krakow, Poland; 5 RECETOX, Faculty of Science, Masaryk University, Kotlarska 2, Brno, Czech Republic; 6 Loschmidt Laboratories, Department of Experimental Biology, Faculty of Science, Masaryk University, Kamenice 5, Brno, Czech Republic; 7 International Clinical Research Center, St. Anne’s University Hospital Brno, Pekarska 53, Brno, Czech Republic; 8 Department of Pharmacology and Toxicology, Faculty of Pharmacy, Charles University, Heyrovskeho 1203, Hradec Kralove, Czech Republic

**Keywords:** Cytochrome P450, Salidroside, Rhodioloside, Metabolic activity, Drug-herb interactions

## Abstract

**Background:**

Salidroside (SAL) is the active ingredient of the traditional adaptogenic herb *Rhodiola rosea*. Cytochromes P450 (P450), crucial enzymes in drug metabolism, are central to understanding drug–herb interactions. This study investigates the impact of SAL on the metabolic activity of selected P450 in both rat and human systems.

**Methods:**

Wistar rats were administered intragastrically with SAL 5, 15, or 45 mg/kg/day for seven days. The metabolic activity of CYP1A2, CYP2C6, CYP2D, and CYP3A was measured in rat liver microsomes (RLMs). Amounts and gene expressions of CYP1A2 and CYP2C6 were assessed in RLMs using Western blot and two-step qRT-PCR. rPXR, hPXR, and CAR3 gene reporter assays were conducted. The in vitro inhibitory studies of SAL in both drug-naïve rat and human liver microsomes were performed. Interactions between SAL and human P450 were also studied by in silico methods.

**Results:**

SAL at the dose of 5 mg/kg/day slightly increased the specific activity of the P450 studied. However, SAL did not change either the P450 protein levels or the expression of the corresponding genes in rat liver. It also did not cause direct inhibition of rat and human P450 in liver microsomes in vitro. Molecular docking with human P450 confirmed these findings. Moreover, SAL inhibited the agonist-mediated induction of both rat and human PXR.

**Conclusions:**

Based on our findings, SAL is unlikely to pose a significant risk of P450-mediated drug-herb interactions and rather preserves the constitutional CYP3A metabolic activity via interaction with PXR.

**Clinical trial number:**

Not applicable.

**Supplementary Information:**

The online version contains supplementary material available at 10.1007/s43440-026-00842-w.

## Introduction

 Phytotherapy remains an important component of contemporary healthcare, particularly within the context of complementary and alternative medicine. The World Health Organization (WHO) estimates that approximately 60% of the global population uses herbal medicinal products, while nearly 80% of individuals in developing countries rely largely on these therapies to address their primary healthcare needs [1]. In parallel, the use of phytotherapeutic products is increasing worldwide [2]. This growing interest may be partly attributed to concerns about the adverse effects of synthetic pharmaceuticals, leading to the widespread perception that herbal preparations are safer alternatives [3]. Surveys revealed that about 50% of consumers do not believe natural medications cause side effects and do not report their use to physicians [4]. Almost 90% of patients over 50 years reported concomitant use of conventional and complementary therapies [5].

*Rhodiola rosea (Crassulaceae)* is an herb traditionally used in northern countries of Europe, Asia, and North America for its adaptogenic and strengthening properties. The main phytochemicals contained are salidroside (SAL), rosavin, rosin, rosarin, and tyrosol. However, more than 109 compounds were isolated [6]. The European Union herbal monograph on *Rhodiola rosea L.*,* rhizoma et radix* summarises its effects as the “relief of symptoms of stress, such as fatigue and exhaustion” [7]. Pharmacological effects of the herb, including antidepressant, antistress, antifatigue, antidiabetic, or anticancer activities, are documented in both preclinical and clinical trials [6].

SAL (2-(4-hydroxyphenyl)ethyl-beta-D-glucopyranoside, rhodioloside), is one of the main active substances of *R. rosea* [8] responsible for its beneficial effect. The antidepressant-like effects of SAL have been reported in various animal studies. The 2-week treatment with doses of 20–40 mg/kg/day significantly decreased depressive-like behaviour in rats [9]. Similarly, in the mouse model of depression, either a 5-day pre-treatment with 12 and 24 mg/kg/day of SAL [10] or 14 days of SAL administration at a dose of 10 mg/kg [11] had antidepressant-like activity. Chai et al. also proved that SAL at doses of 20 and 40 mg/kg has comparable antidepressant-like effects with fluoxetine (20 mg/kg) in two mouse models of depression [12]. Besides the above-mentioned, recent reviews described in detail the therapeutic effects of SAL on cardiovascular diseases [13–16] and its anticancer properties [17]. Therefore, SAL is a promising candidate for complementary or alternative medicine of herbal origin.

The pharmacokinetics of SAL is described in detail elsewhere [16, 18–21]. After intravenous application, SAL is distributed mainly into the liver, fat, and skeletal muscle [20]. Gut absorption is dependent on limited active transport via sodium-dependent glucose cotransporter 1 (SGLT1) [16]. In rats, the AUC_0-∞_ was 1370 ng·h/mL after oral administration of 0.57 mg/kg [22], the bioavailability varied between 52 and 98% after intragastric and oral application [23, 24] and the elimination half-life was approximately 1.3 h after intragastric administration [24]. Glucuronidation, sulfation, and deglycosylation to p-tyrosol are the major metabolic pathways of SAL followed by renal excretion [20, 25].

Cytochromes P450 (P450) constitutes a complex of hemoprotein monooxygenase enzymes located mainly in the liver, estimated to metabolise most of the medications used in clinical practice. The alteration of P450 metabolic activity by drugs or substances of herbal origin may occur in clinical practice and is a common mechanism of drug-herb interactions [26]. It may result in severe adverse effects or the failure of patients’ pharmacotherapy. Serious consequences of the concomitant use of herbal constituents with P450 substrates can be found elsewhere [27]. Contrary to standard medicinal products that undergo testing for possible interactions with P450 before being introduced to clinical practice, herbal supplements often lack this information.

Our study primarily aimed to evaluate the effect of the promising herbal antidepressant SAL on the most important P450 isoforms. Different experimental models were used to study the potential influence of SAL on P450 metabolic activity and its mechanism. Therefore, we gained complex knowledge about the risk of drug-SAL interaction via P450 enzymes. Secondly, possible biochemical and haematological marks of SAL toxicity in rats were monitored.

## Materials and methods

SAL (purity 99.49%, Lot No. AF2020721) was purchased from Chengdu Alfa Biotechnology (PRC). 1´-OH bufuralol, 4´-hydroxydiclofenac, α-naphthoflavone (catalog number: N5757), acetaminophen (catalog number: A7085), caffeine, citric acid, dextromethorphan (catalog number: 81091), dextrorphan (catalog number: D127), diclofenac (catalog number: D6899), EDTA, glucose, glucose-6-phosphate dehydrogenase, HCl, HClO_4_, chlorpropamide (catalog number: C1290), ibuprofen (catalog number: I4883), ketoconazole (catalog number: K1003), KH_2_PO_4_, K_2_HPO_4_, laudanosine (catalog number: L1389), MgCl_2_. 6 H_2_O, Na_2_CO_3_, Na_2_HPO_4_, NaH_2_PO_4_, NH_3_, NH_4_HCO_3_, paraxanthine, phenacetin (catalog number: 2375), progesterone (catalog number: P8783), quinine (catalog number: Q1125), sodium dithionite, sucrose, testosterone (catalog number: 86500), triethylamine, Tris and ZnSO_4_ were ordered from Sigma-Aldrich (USA). Acetonitrile, diethyl ether, dichloromethane, ethyl acetate, hexane, methanol and propane-2-ol (used for in vitro experiments at the Department of Pharmacokinetics and Drug Metabolism, Maj Institute of Pharmacology) were purchased from Merck (GER). Acetic acid, KCl and n-butanol were obtained from Honeywell (USA). Diethyl ether, ethanol 96% and propylene glycol were ordered from Fagron (CZE), acetonitrile, hexane and methanol from VWR (USA), N-desmethylperazine and perazine from Labor (PL), fluoxetine from Orion (FIN), dichlormethane from Penta (CZE) and glucose-6-phosphate with NADPH from Carl Roth (GER). 6β-hydroxytestosterone (catalog number: A5735-000) was purchased from Steraloids (USA).

### In vivo animal experiment

#### Animals and experimental design

Male eight-week-old Wistar albino rats (*n* = 40) were purchased from the Masaryk University breeding facility (CZE). Animals were housed in plastic cages in standard laboratory conditions (temperature 22 ± 2 °C, humidity 55 ± 5%, 12-hour light-dark cycle, mean weight 242.2 ± 13.2 g) with access to food and water *ad libitum*. All experiments were performed according to the Czech Act. No 246/1992, Coll and Directive 86/609/EEC, and were approved by the Expert committee for ensuring the welfare of experimental animals of the Faculty of Medicine and national Central Commission for Animal Welfare (MSMT-46285/2020-3).

Rats were acclimatised for four days. On the 5th day, they were randomly divided into four groups of 10 animals. Three groups were administered intragastrically with an aqueous solution of SAL (2.5, 7.5 and 22.5 mg/mL of SAL completely dissolved in demineralised water, the fresh solutions were prepared daily) at 5, 15, or 45 mg/kg of body weight/day for seven consecutive days (SAL5, SAL15, and SAL45). The control group (C) was administered the appropriate volume of water (2 mL/kg/day). The selection of doses of SAL was based on the documented antidepressant effect of SAL observed in in vivo rat models [9, 28]. To eliminate the influence of circadian variation in *P450* gene expression, all experimental groups were divided into halves with a one-day shift in the experimental design. The sample harvesting was performed in all animals between 8 and 11 a.m. for two subsequent days by decapitation under ether anaesthesia. Liver samples were drawn 24 h after the last SAL administration were weighed and frozen at -80 °C until microsomal and mRNA extraction.

#### Haematological and biochemical parameters

To evaluate the possible toxic effects of the SAL in vivo, general haematological and biochemical markers and total body and liver weight were assessed. Basic haematological and biochemical parameters were analysed at the Small Animal Clinic Laboratory at the University of Veterinary Sciences Brno (CZE).

The blood samples were collected into the 2 mL test tubes with EDTA and white blood cell, platelet and red blood cell count, haemoglobin and haematocrit levels, mean corpuscular volume (MCV), mean corpuscular haemoglobin (MCH), mean corpuscular haemoglobin concentration (MCHC), red blood cell distribution width (RDW), platelet distribution width (PDW), mean platelet volume (MPV), and plateletcrit (PCT) were analysed on Sysmex XT-2000iV (Sysmex, JPN).

Other blood samples were collected in the 2 mL test tubes with Li-heparin and centrifuged at 1500 x g for 10 min at 20 °C. Total protein, albumin, and total bilirubin content, alanine transaminase (ALT), aspartate transaminase (AST), alkaline phosphatase (ALP), and gamma-glutamyl transferase (GGT) were biochemical parameters measured on the Architect c4000 (Abbott, USA).

The animals’ body weights were monitored daily at the same time before SAL administration. Average body weight and average weight gain during SAL treatment were compared between groups.

#### Isolation of rat liver microsomes, total protein, and total P450 content

Rat liver microsomes (RLMs) were isolated by the differential ultracentrifugation method described in detail in our previous study [29]. One sample from the C group was lost during the process of isolation. The total protein content in RLMs was evaluated by Pierce™ BCA Protein Assay Kit (Thermo Fisher Scientific, USA) according to the manufacturer’s protocol. P450 and cytochrome P420 content was measured using Omura and Sato’s CO difference spectroscopy method [30].

#### Determination of P450 metabolic activity in isolated RLMs

The metabolic activity of CYP1A2, CYP2C6, CYP2D, and CYP3A in isolated RLMs was assessed as a rate of conversion of specific substrates by P450 in the presence of the NADPH-generating system according to the modified method published previously [31].

The conditions of reactions were tested for the linear range (data not shown). The reactions were started by adding RLMs (1 mg of protein/1 mL in the total volume of 0.5 mL of sample) to the incubation mixture, stopped by 100 µl of cold acetonitrile, and placing the sample on ice. Each sample was prepared in triplicate. The amounts of metabolites were assessed using either HPLC Dionex Ultimate 3000RS LC (Thermo Fisher Scientific, USA) with a DAD detector (CYP1A2, CYP2C6, and CYP3A) or Shimadzu LC-10 (Shimadzu, JPN) with a fluorescence detector (CYP2D). The specific (amount of metabolite formed per mg of protein per min) and molecular (amount of metabolite formed per nmol of P450 per min) activities were calculated.

To assess the metabolic activity of P450, the rate of phenacetin O-deethylation [29], diclofenac 4´-hydroxylation [32], dextromethorphan O-demethylation [29], and 6β-testosterone hydroxylation [33] were used as described previously. The minor modifications of the methods are summarised in Table 1.

#### Western blot analysis

Based on the findings of a significant increase in the specific metabolic activities of CYP1A2 and CYP2C6, we further evaluated the protein and mRNA expression levels of these isoforms in RLMs. For Western blotting, slightly modified methods described elsewhere were used [29]. Diluted samples of control animals were pooled. Primary and secondary antibodies were diluted according to Table 2. The protein levels of loading control (β-actin) differed significantly between animals harvested on separate days. Therefore, two sets of pooled controls were used for Western blot analyses, and samples from SAL-treated groups were compared to controls on the adequate day of sample harvesting. Western blot analysis of each sample was performed in technical triplicate. Results have been presented as a relative amount of protein (% of controls) per nmol of total P450 content.

**Table 1:** P450 metabolic activity – incubation and analytical conditions; CZE – Czech Republic, USA – United States of America

P450	1A2	2C6	2D	3 A
Substrate	Phenacetin	Diclofenac	Dextromethorphan	Testosterone
Substrate concentration [µM] [29]	400	100	500	400
Incubation time [min]	20	20	20	15
Metabolite	Acetaminophen	4’- hydroxydiclofenac	Dextrorphan	6β-testosterone
HPLC system	Dionex Ultimate 3000RS LC	Dionex Ultimate 3000RS LC	Shimadzu LC-10	Dionex Ultimate 3000RS LC
Column	Accucore PFP^1^	Accucore C18^1^	Tessek Phenyl^2^	Accucore C18^1^
Column oven [°C]	35	35	room temperature (22 °C +- 3 °C)	40
Mobile phase A	0.1% NH_4_CH_3_COO, pH 4.6	10 mM NH_4_HCO_3_, pH 7.1 (acetic acid)	20 mM KH_2_PO_4_ with 0.1% triethylamine, pH 3.8	1% methanol
Mobile phase B	acetonitrile			
Flow [mL/min]	0.4	0.75	0.65	0.9
Gradient (A: B)	0 min 90:10 5 min 35:65	0 min 85:15	isocratic flow 50:50	0 min 17:83
	5–7 min 35:65	4 min 79:21		10.5 min 19:81
	7–10 min 90:10	5.5 min 60:40		13.8 min 45:55
		5.7–6.5 min 50:50		14 min. 60:40
		6.7–8 min 82:18		16 min. 40:60
				16.2–19 min 15:85
Sample processing	L: L^3^ diethyl ether	centrifugation, 10 min., 13 000 x g	L: L^3^ n-butanol: hexan 1:9	L: L^3^ dichloromethane
Detection	UV 245 nm	UV 276 nm	fluorescence detection λ ex 280 nm, λ em 320 nm	UV 254 nm

^1^ Accucore columns (100 × 3 mm, 2.6 μm, Thermo Fisher Scientific, USA) with pre-column

^2^ Tessek column – Tessek phenyl (150 × 3 mm, 5 μm, Tessek, CZE)

^3^ L: L, liquid: liquid extraction + evaporation under gentle N_2_ stream

**Table 2:** The properties of primary and secondary antibodies used in Western blot analysis

Antibody	Company and catalogue No.	Biological source	Species	Dilution
Anti-CYP1A	Santa Cruz Biotechnology (sc-53241)	Mouse	Mouse, rat, human	1:1000
Anti-CYP2C6	Santa Cruz Biotechnology (sc-73484)	Mouse	Mouse, rat, human	1:2000
Anti- β-actin	Cell Signalling Technology (cs-4970)	Rabbit	Mouse, rat, human, monkey, bovine, pig	1:2000
Anti-mouse	Sigma-Aldrich (A4416)	Goat	Mouse	1:5000
Anti-rabbit	Sigma-Aldrich (A0545)	Goat	Rabbit	1:5000

#### Analysis of mRNA by real-time quantitative reverse transcription polymerase chain reaction

The expressions of the *CYP1A2* and *CYP2C6* genes in the liver samples were investigated in all experimental groups. Three bioptic samples (approx. 30 mg per sample) from different liver sites were obtained from each animal 24 h after the last administration of SAL. They were immediately frozen at -80 °C until RNA extraction and then evaluated by technical duplicate using the two-step real-time quantitative reverse transcription polymerase chain reaction (qRT-PCR) method.

Liver samples were thawed entirely on wet ice and homogenised using Precellys Evolution homogeniser (Bertin Technologies, FRA) and ceramic beads 1.4 mm (Qiagen, GER). Total RNA was extracted using the Total RNA Mini kit (catalog numbers: 031 − 25, 031–100, A&A Biotechnology, PL). The quality and quantity of isolated RNA were determined by measuring the absorbance and then fluorescence using Quant-iT™ RNA Assay Kit, Broad Range (catalog number: Q10213, Thermo Fisher Scientific, USA), respectively, both on the Synergy HTX Multimode Reader (Agilent, USA). Moreover, the quality of extracted RNA was analysed by determining the RNA integrity number (RIN) on the Fragment Analyzer (Agilent, USA) in 8 randomly selected samples. The average RIN value of 8 reference samples was 7.46 ± 1.22, proving the good quality of isolated RNA [34]. For further analyses, we aimed to obtain RNA with a purity ratio of 260/280 > 1.80; however, two samples had lower quality.

Reverse transcription of RNA template (input of total RNA was 1 µg) to cDNA was performed using Transcriptor First Strand cDNA Synthesis Kit (catalog number: 04897030001, Roche Diagnostics, GER) with random hexamer primers according to the manufacturer’s protocol; two types of negative controls (I. PCR grade water without RNA; II. PCR grade water with RNA but without reverse transcriptase) were used. For the amplification and detection of cDNA, the FastStart TaqMan^®^ Probe Master (catalog number: 06402682001, Roche Diagnostics, GER) and three TaqMan Gene Expression Assays (ThermoFisher Scientific, USA), which are specific for rat *CYP1A2* (catalog number: 4331182, Rn00561082_m1), *CYP2C6* (catalog number: 4331182, Rn03417171_gH), and *glyceraldehyde-3-phosphate dehydrogenase*(GAPDH; catalog number: 4331182, Rn01775763_g1), were used. The qPCR was carried out in technical duplicates on LightCycler^®^ 480 Instrument (Roche Diagnostics, USA) according to the manufacturer’s protocol; the WB-F344 Rat Liver (James E. Trosko, Michigan State University, USA) [35] was chosen as an interplate control. Both negative and positive controls (cell line with appropriate assay – *CYP1A2*, *CYP2C6*, or *GAPDH*) were also included. Using a relative quantification approach, the output data were analysed using 7500 SDS software (version 1.41, Thermo Fisher Scientific, USA; LightCycler^®^ 480 SW 1.5.1). The relative expression of the targeted genes was determined by the ΔΔCt method and normalised to the expression of the endogenous housekeeping control gene – *GAPDH* [36].

### In vitro inhibitory assays of SAL with P450 enzymes

The experiments were performed in accordance with standard operating procedures of each laboratory.

#### Rat liver microsomes

The incubations of pooled drug-naïve RLMs (0.25 mg of protein/mL of a total volume of 1 mL of sample) were performed to evaluate the direct interaction between SAL and P450 enzymes. RLMs were isolated from ten 16-week-old, drug-naïve male Wistar albino rats and were pooled for the inhibition studies. The activities of the same P450 as in the in vivo model were assessed. The concentrations of specific substrates were set to the Km values, and the incubation times were prolonged but still to be in the linear range as follows: phenacetin 210 µM for 40 min, diclofenac 8 µM for 30 min, testosterone 100 µM for 20 min, dextromethorphan 28 µM for 40 min. Tested concentrations of SAL in samples were 100 µM, 10 µM, 1 µM, and 0.1 µM. The effect of preincubation of SAL with RLMs for 10 min before adding a specific substrate was evaluated. The model inhibitors α-naphthoflavone, fluoxetine, quinine, and ketoconazole at the concentrations of 2 nM-20 µM were used for CYP1A2, CYP2C6, CYP2D, and CYP3A inhibitions, respectively [37, 38]. Each sample was prepared in triplicate. The buffer composition containing the NADPH-generating system, sample processing, and analytic methods were identical to those mentioned above. The reaction rate was calculated, and the results have been presented as a % of the control activity (mean reaction rate in SAL-treated samples divided by the mean reaction rate in control samples, multiplied by 100).

#### Human liver microsomes

Pooled human liver microsomes (HLMs; 0.5 mg of protein/1 mL of sample volume; BD Biosciences Discovery Labware, USA) were incubated with the NADPH-generating system, SAL (final concentrations of 500 µM, 250 µM, 100 µM, 50 µM, 10 µM, 1 µM) or with vehicle (control samples). The incubations were started by adding HLMs. Each sample of the final volume of 0.5 mL was prepared in triplicate. Caffeine N-demethylation (CYP1A2), 4´-hydroxylation of diclofenac (CYP2C9), N-demethylation of perazine (CYP2C19), bufuralol 1´-hydroxylation (CYP2D6), and 6β-hydroxylation of testosterone (CYP3A4/5) were used. Concentrations of specific substrates and further incubation conditions were as previously described [39]. The experiments were performed at the Department of Pharmacokinetics and Drug Metabolism, Maj Institute of Pharmacology. The reaction rate was calculated, and the results have been presented as a % of the control activity (mean reaction rate in SAL-treated samples divided by the mean reaction rate in control samples, multiplied by 100).

### Nuclear receptor gene reporter assays

The nuclear receptor gene reporter assay was used to evaluate the modulatory effects of SAL on the rat and human pregnane X receptor (PXR) and human constitutive androstane receptor type 3 (CAR3). HepG2 cells were transfected with a luciferase reporter construct, p3A4-luc, and either a rat PXR expression vector (pCMV-rPXR) or a human PXR expression vector (pSG5-hPXR B). The CAR3 variant (XM_005245697.4, transcript variant X4; engineered to exhibit reduced basal activity) was co-transfected with the CYP2B6-luc reporter plasmid (originally designated B-1.6k/PB/XREM). In all assays, the pRL-TK Renilla luciferase vector was included for normalization. Cells were treated with the rat PXR agonist pregnenolone-16α-carbonitrile (PCN), the human PXR agonist rifampicin (Rif), or the CAR agonist 6-(4-chlorophenyl)imidazo[2,1-b] [1,3]thiazole-5-carbaldehyde O-(3,4-dichlorobenzyl)oxime (CITCO), alone or in combination with SAL at 5 and 10 µM, for 24 h. Firefly and Renilla luciferase activities were measured using the Dual-Glo^®^ Luciferase Assay System (catalog number: E1960, Promega, Madison, USA) [40]. Data are presented as relative activation compared to the dimethyl sulfoxide (DMSO) control (set as 1). Results represent the mean ± standard error of the mean of at least three independent experiments.

### Molecular docking study

#### Protein selection and preparationss

We obtained all protein crystal structures from RCSB PDB [41], but only those of human origin were further analysed due to the absence of rat P450 structures in the database. The selected protein structures for each P450 target, along with their corresponding PDB IDs, inhibitors and mapping to the rat orthologs, are displayed in Table 3, along with their corresponding PDB IDs, inhibitors, and mappings to rat orthologs.

**Table 3:** Selected PDB structures of human P450 proteins, their inhibitors, and rat orthologs; P450 – cytochrome P450

Human	Rat	Inhibitor	PDB code
CYP1A2	CYP1A2	Furafylline	2HI4
CYP2C9	CYP2C11	Sulfaphenazole	1R9O
CYP2D6	CYP2D1/2	Paroxetine	3TBG
CYP3A4/5	CYP3A1	Ketoconazole	1TQN

To ensure precise binding site geometry, we prioritised crystal structures with high resolution. Furthermore, we chose structures that contain inhibitors of a similar size range to SAL. This selection process helps prevent potential inaccuracies arising from overly tight or loose binding sites.

Although a crystal structure of CYP3A4/5 with ketoconazole is available (PDB ID 2V0M), it wasn’t suitable for our study due to its excessively enlarged cavity, which accommodates two inhibitor molecules, each with a higher molecular mass (530 Daltons) than SAL (300 Daltons). Therefore, we decided to use the PDB structure of CYP3A4/5 (1TQN), which doesn’t have a ligand but has a cavity of the right size to fit SAL and shows high resolution (2.05 Å).

Except for heme groups, the PDB files were processed to remove water molecules, ions, and ligands. The H + + webserver [42] was used to add hydrogen atoms to the chosen apoprotein structures. The heme group parametrisation was performed using a Python-based Metal Center Parameter Builder (MCPB.py) [43]. Protein hydrogen atoms were optimised using the AMBER [44] software with the ff14SB [45] force field in the IGB7 [46] implicit solvent model environment to avoid clashes.

#### Ligand preparations

We obtained ligands from ChemSpider [47] in SMILES format. We generated their 3D geometry using the LigPrep [48] module with Optimised Potentials for Liquid Simulations (OPLS3e) force field [49]. Epik [50] implemented in LigPrep was used for calculating protonation and tautomerisation states at pH 7.0 ± 2. Ligands with a state-penalty value between 0 and 1 were selected for further docking calculations and scoring steps. The prepared ligand geometries were optimised using MOPAC2016 [51] software at PM6-D3H4X [52] level with the implicit solvent model COSMO [53]. The GAFF2 [54] was used to parametrise ligand geometries in the antechamber module of AMBER software. Partial atomic charges were calculated at the DFTB3 [55] level.

#### Molecular docking

The Protein-Ligand ANT System (PLANTS) docking software [56] was used to generate poses, employing the chemplp scoring function. The docking centre was positioned approximately 2.3 Å above the iron atom of the heme group. Docking radii of 15 Å, 15 Å, 10 Å, and 15 Å were used for 1R9O, 1TQN, 2HI4, and 3TBG, respectively, to include the entire inner catalytic cavity and access tunnels. We generated at least 100 poses per ligand and increased the number of poses by 10 for each rotatable bond in the ligand structure. Protein flexibility was not considered during the docking process.

#### Geometry optimizations and clustering

The AMBER software’s LEaP function was used to create complex geometries of ligand conformations. Only the ligand atoms and protein hydrogen atoms within 4 Å of all docked ligand poses were allowed to move. An initial molecular mechanics energy minimisation step was applied *in vacuo* using the ff14SB force field to eliminate clashes and quickly form complementary interactions. An RMSD-based clustering approach was employed to reduce the number of docking poses, thereby decreasing the computational cost for subsequent, more demanding minimisation calculations with implicit solvent. The clustering process used an RMSD cutoff of 1 Å and selected the highest-scoring pose from each cluster. A final step of molecular mechanics minimisation was applied with IGB7 solvation to calculate the score, accounting for desolvation upon interactions. The Cuby4 computational chemistry framework [57] was used for the optimisation and clustering calculations.

#### Scoring

We estimated the affinity of ligands to P450 proteins using a scoring function (Eq. 1) that combines the protein-ligand interaction energy in implicit solvent (E_interaction_) with the conformational free energy changes of the ligand (ΔG_conf_(ligand)) and protein hydrogen atoms (ΔG_conf_(Protein_H_)) upon complexation.

Equation 1: Calculation of the scoring function for the affinity of P450 ligands

1 $$\eqalign{ \>Score = & {E_{interaction}} + \>\Delta \>{G_{conf}}\left( {ligand} \right)\> \cr & + \>\Delta \>{G_{conf}}\left( {Protei{n_H}} \right) \cr} $$

### Statistics and data presentation

Data distribution was assessed using histograms and Shapiro-Wilk tests, revealing most data to be non-normally distributed. Consequently, the statistical significance of the effects of SAL or vehicle administration in vivo was evaluated using the non-parametric Kruskal-Wallis test, followed by multiple comparisons for total protein content, total P450 content, cytochrome P420 content, specific and molecular metabolic activities, and mRNA levels. The differences in CYP1A2 and CYP2C6 content (Western blot) between experimental groups and controls were evaluated by the Wilcoxon single-rank test followed by Bonferroni correction. Statistica software (version 14.0.0.15, TIBCO Software Inc., USA) was used for statistical evaluation. For PXR gene reporter assays, statistical significance was determined using ordinary one-way ANOVA followed by Tukey’s multiple comparison test, performed with GraphPad PRISM (version10.4.0) (621) GraphPad Software, Boston, USA). Results were considered statistically significant when *p* ≤ 0.05.

The data obtained within this experiment are available on the Mendeley data [58].

## Results

### In vivo animal experiment

#### Haematological and biochemical parameters, body and liver weight

We did not detect any signs of toxicity of orally administered SAL to rats for seven days at doses ranging from 5 to 45 mg/kg. The average total serum protein, albumin and total bilirubin levels in the C group were 58.24 ± 2.07 mg/mL, 31.18 ± 1.01 mg/mL, and 1.90 ± 0.16 µmol/L, respectively. None of the evaluated biochemical parameters differed in the SAL-treated animals from the values found in the C group.

Similarly, none of the evaluated haematological parameters differed between controls and SAL-treated animals regardless of the administered SAL dose.

The average weight of the animals at the beginning of the experiment did not differ between the experimental groups and ranged from 271 to 289 g. The average daily weight gain during the investigation was between 5.6 and 6.6 g/day. Finally, the average weight of harvested livers ranged between 9.43 g and 11.12 g. It did not differ between SAL-treated rats and control animals, confirming the hepatic safety of SAL use.

#### Total protein and P450 content in isolated RLMs

The median (min. – max.) concentration of total protein in isolated RLMs from C group was 16.86 (10.19–19.19) mg/mL, while in the SAL5, SAL15 and SAL45 groups, they were 15.93 (11.82–23.34) mg/mL, 16.83 (11.76–21.09) mg/mL, and 20.45 (12.56–22.18) mg/mL, respectively. No statistically significant difference in this parameter among the studied groups was found.

**Fig. 1:**
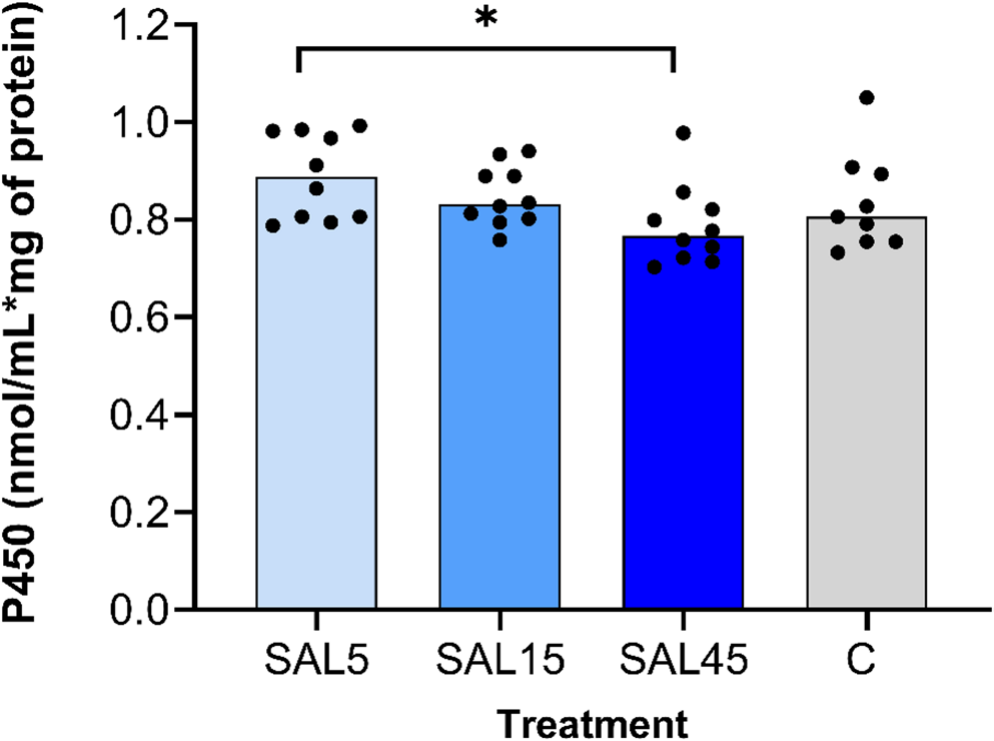
Changes in P450 content in RLMs isolated from rats treated intragastrically for seven days with SAL at the doses of 5 mg/kg/day (SAL5, *n* = 10), 15 mg/kg/day (SAL15, *n* = 10), 45 mg/kg/day (SAL45, *n* = 10) or with water (C, *n* = 9). The P450 content was obtained with CO difference spectroscopy according to the modified method of Omura and Sato [30]. Each sample was prepared in technical triplicate. Statistical significance was assessed by the Kruskal-Wallis statistical test followed by multiple comparison tests. Columns represent medians, each data point represent the biological replicates. *p < 0.05 versus SAL45 group. C – control group, P450 – cytochrome P450, RLMs – rat liver microsomes, SAL – salidroside

A significant difference (Kruskal-Wallis test, H = 7.88, *p* = 0.049, N_1_ = 10, N_2_ = 10, N_3_ = 10, N_4_ = 9) was found in the concentration of P450 among groups. *Post hoc* analysis determined an increased P450 level in the SAL5 group compared to SAL45 animals. No differences were found between the SAL-treated and the C groups (Fig. 1). The P450 content per g of liver tissue and cytochrome P420 content did not differ between groups.

These differences between SAL groups indicate that specific activity that compares the activity of the same amount of protein should vary between these groups because the content of P450 in incubation mixtures will be different. This difference should be minimised in the case of molecular activity, where the activities of the same amount of P450 are compared, or activity calculated by g of liver tissue, where a decreased amount of P450/mg of protein in SAL45 could be compensated by increased total protein content.

#### Determination of P450 metabolic activity in isolated RLMs

**Fig. 2:**
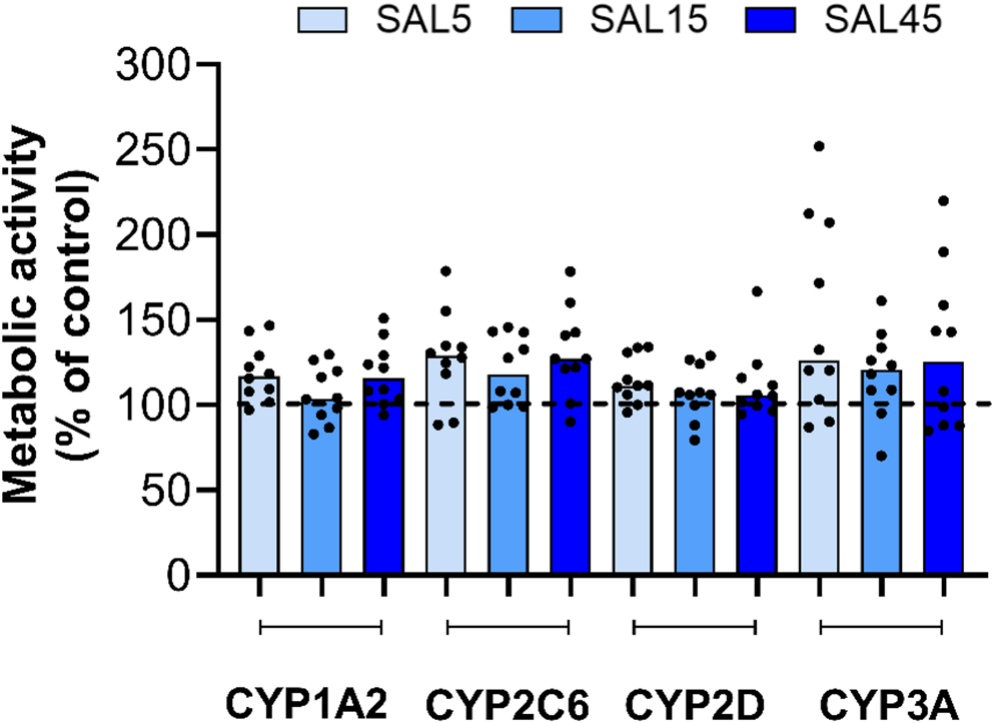
Molecular metabolic activities (of the same amount of P450) of CYP1A2, CYP2C6, CYP2D, and CYP3A measured as the rate of phenacetin O-deethylation, diclofenac 4´-hydroxylation, dextromethorphan O-demethylation and 6β-testosterone hydroxylation, respectively, in RLMs isolated from rats treated intragastrically with SAL for seven consecutive days at the doses of 5 mg/kg/day (SAL5, *n* = 10), 15 mg/kg/day (SAL15, *n* = 10), 45 mg/kg/day (SAL45, *n* = 10), expressed as a per cent of median metabolic activity of the C treated with water (*n* = 9). Reactions were performed in the presence of 400 µM phenacetin (CYP1A2), 100 µM diclofenac (CYP2C6), 500 µM dextromethorphan (CYP2D) and 400 µM testosterone (CYP3A) in RLMs (1 mg/ml of the total protein content) with an NADPH-generating system in a final volume of 0.5 ml at 37 °C. Each sample was prepared in technical triplicate. Columns represent medians, each data point represent the biological replicates. The median (min. – max.) values of C for CYP1A2, CYP2C6, CYP2D, and CYP3A were as follows: 1.41 (1.20–1.85), 0.34 (0.25–0.46), 0.92 (0.70–1.21), and 0.38 (0.20 − 0.61) nmol/min/nmol of P450. Statistical significance was evaluated with Kruskal-Wallis statistical test followed by multiple comparison test of mean ranks versus the C. C – control group, P450 – cytochrome P450, RLMs – rat liver microsomes, SAL – salidroside

**Fig. 3:**
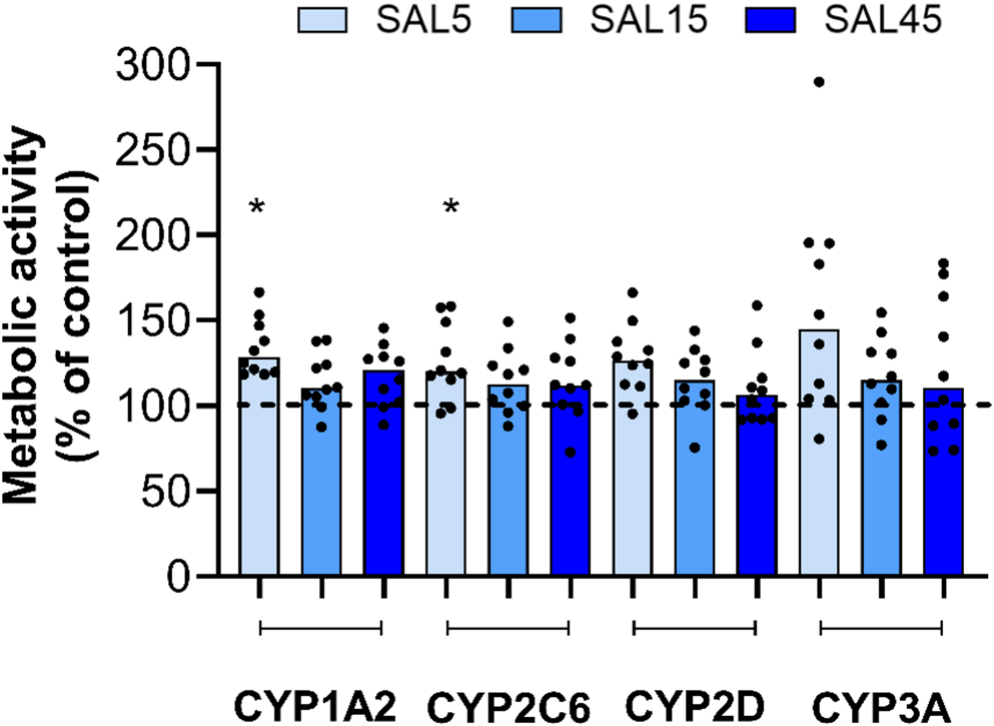
Specific metabolic activities (of the same amount of protein) of CYP1A2, CYP2C6, CYP2D, and CYP3A measured as the rate of phenacetin O-deethylation, diclofenac 4´-hydroxylation, dextromethorphan O-demethylation and 6β-testosterone hydroxylation, respectively, in RLMs isolated from rats treated intragastrically with SAL for seven consecutive days at the doses of 5 mg/kg/day (SAL5, *n* = 10), 15 mg/kg/day (SAL15, *n* = 10), 45 mg/kg/day (SAL45, *n* = 10), expressed as a per cent of median metabolic activity of the C treated with water (*n* = 9). Reactions were performed in the presence of 400 µM phenacetin (CYP1A2), 100 µM diclofenac (CYP2C6), 500 µM dextromethorphan (CYP2D) and 400 µM testosterone (CYP3A) in RLMs (1 mg/ml of the total protein content) with an NADPH-generating system in a final volume of 0.5 ml at 37 °C. Each sample was prepared in technical triplicate. Columns represent medians, each data point represent the biological replicates. The median (min. – max.) values of the C for CYP1A2, CYP2C6, CYP2D, and CYP3A were as follows: 1.11 (0.90 − 1.65), 0.30 (0.21–0.35), 0.74 (0.58–1.02), and 0.32 (0.17–0.46) nmol/min/mg of protein. Statistical significance was evaluated with Kruskal-Wallis statistical test followed by multiple comparison test of mean ranks versus the C and is indicated with *p < 0.05. C – control group, P450 – cytochrome P450, RLMs – rat liver microsomes, SAL – salidroside

Molecular activities of CYP1A2, CYP2C6, CYP2D, and CYP3A in RLMs isolated from SAL-treated rats were not different from the metabolic activities of controls (Fig. 2), while significant increases in the specific activities of CYP1A2 (Kruskal-Wallis test H = 9.03, *p* = 0.029, N_1_ = 10, N_2_ = 10, N_3_ = 10, N_4_ = 9) and CYP2C6 (Kruskal-Wallis test H = 7.28, *p* = 0.063, N_1_ = 10, N_2_ = 10, N_3_ = 10, N_4_ = 9) in group SAL5 were detected compared to C group (Fig. 3). Based on *post hoc* test, the increase of CYP2C6 metabolic activity was marginally significant (*p* = 0.048).

#### Determination of P450 protein and mRNA levels

**Fig. 4:**
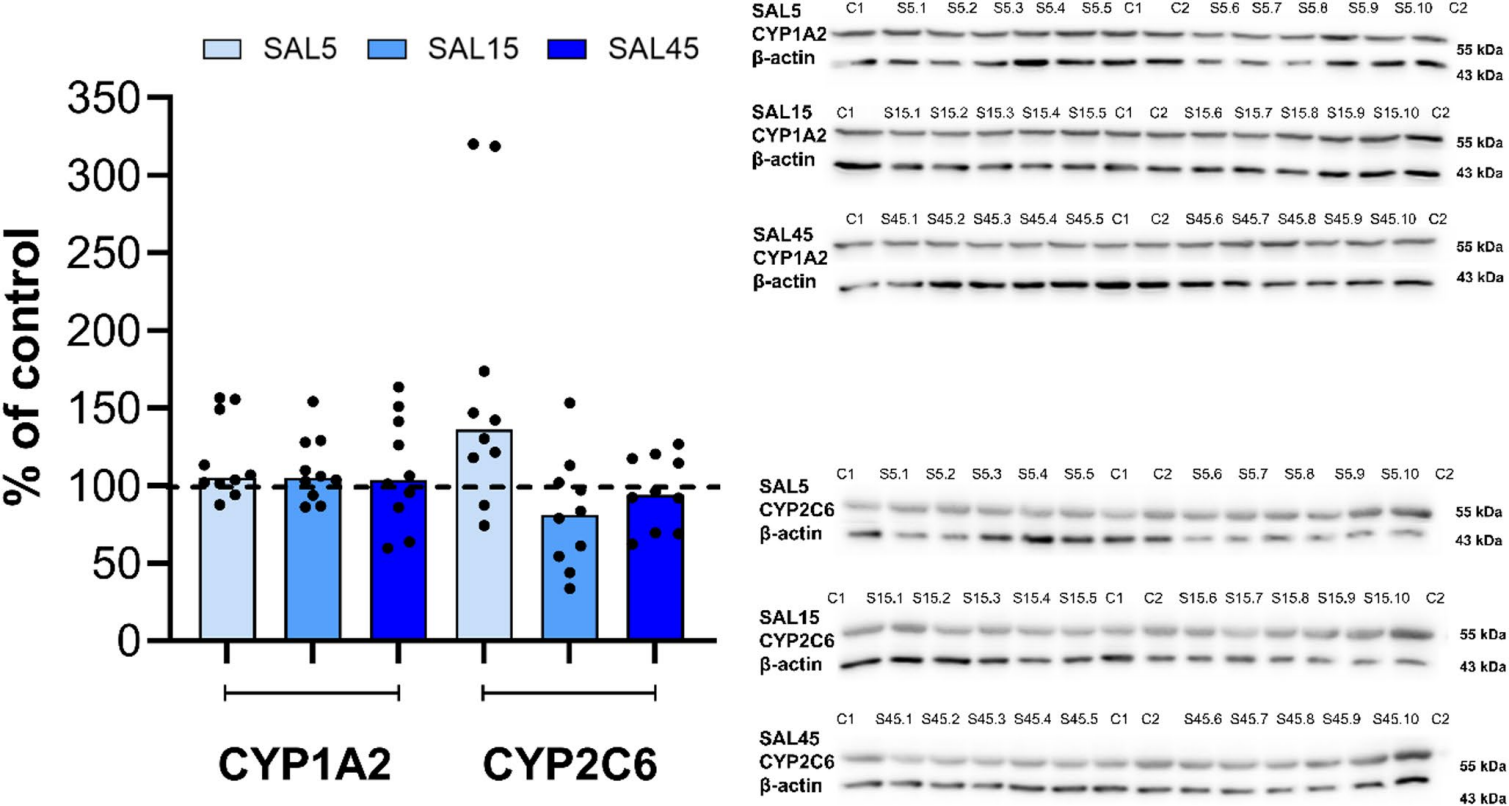
Content of CYP1A2 and CYP2C6 proteins evaluated in samples extracted from rats treated intragastrically with SAL for 7 consecutive days at the doses of 5 mg/kg/day (SAL5, *n* = 10), 15 mg/kg/day (SAL15, *n* = 10), 45 mg/kg/day (SAL45, *n* = 10), expressed as a per cent of the C treated with water (*n* = 9). Each sample was prepared in technical triplicate. Western blots were quantified relative to the loading control (β-actin). The statistical significances were evaluated by the Wilcoxon single rank test followed by Bonferroni correction. Columns represent medians, each data point represent the biological replicates. Representative Western blots show signals of CYP1A2, CYP2C6 and β-actin. Pooled samples of control animals were loaded to the central and edge positions of the gels. The loading control protein levels differed significantly between animals collected on different experimental days. Therefore, two sets of pooled controls were used for Western blot analyses (C1 and C2), and samples from SAL-treated groups were compared to controls on the adequate day of sample harvesting. C – control group, β-actin – beta actin, SAL – salidroside

**Fig. 5:**
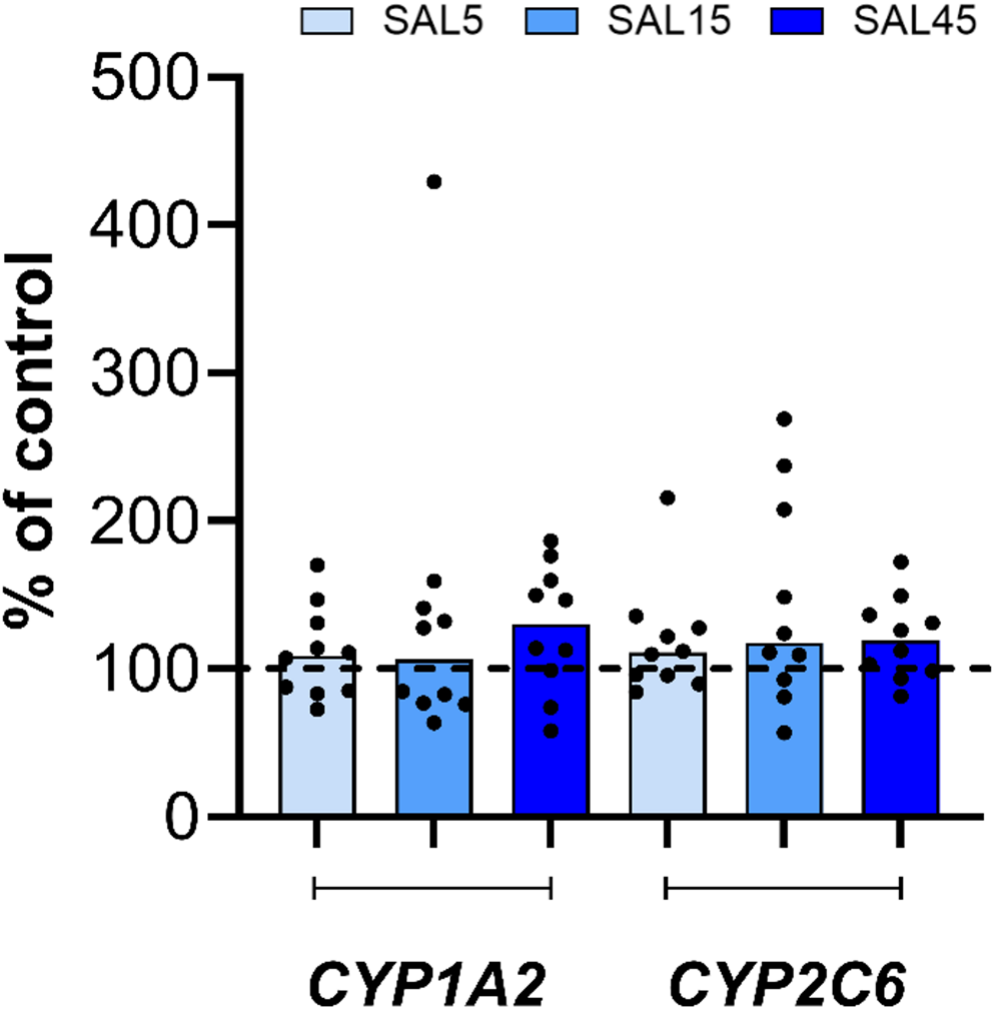
Content of *CYP1A2* and *CYP2C6* mRNAs evaluated in samples extracted from rats treated intragastrically with SAL for 7 consecutive days at the doses of 5 mg/kg/day (SAL5 group, *n* = 10), 15 mg/kg/day (SAL15, *n* = 10), 45 mg/kg/day (SAL45, *n* = 10), expressed as a per cent of the C treated with water (*n* = 9) with qRT-PCR. *GAPDH* was used as endogenous housekeeping control gene. The statistical significances were evaluated with Kruskal-Wallis statistical test followed by multiple comparison test. Columns represent medians, each data point represent the biological replicates. C – control group, GAPDH – glyceraldehyde-3-phosphate dehydrogenase, qRT-PCR – Real-Time Quantitative Reverse Transcription Polymerase Chain Reaction, SAL – salidroside

The concentration of CYP1A2 and CYP2C6 was not affected by SAL administration (Wilcoxon single-rank test – for SAL5: Z = 2.23, *N* = 10, *p* = 0.066 after Bonferroni correction; SAL15: Z = 1.58, *N* = 10, *p* = 0.34 after Bonferroni correction; SAL45: Z = 0.56, *N* = 10, *p* = 1.73 after Bonferroni correction, Fig. 4). Also, *CYP1A2* and *CYP2C6* gene expression was not significantly changed after SAL treatment (Kruskal-Wallis test for *CYP1A2* H = 1.33, *p* = 0.72, N1 = 10, N2 = 10, N3 = 10, N4 = 9, Kruskal-Wallis test for *CYP2C6* H = 1.95, *p* = 0.58, N_1_ = 10, N_2_ = 10, N_3_ = 10, N_4_ = 9; Fig. 5). Therefore, activation of these genes and subsequent increase in amounts of these specific proteins, as well as stabilisation of P450 by SAL preventing their degradation, are probably not involved in the elevated metabolic activity in the SAL5 group.

### In vitro inhibitory assays of SAL with P450 enzymes

The metabolic activities of CYP1A2, CYP2C9, CYP2C19, CYP2D6, and CYP3A4/5 were not significantly changed after incubation of HLMs in the presence of SAL 500 µM.

Similarly, the substrates’ reaction rates in CYP1A2, CYP2C6, CYP2D, or CYP3A were not significantly changed in pooled RLMs from drug-naïve animals in the presence of SAL up to 100 µM. The pre-treatment of RLMs with SAL also did not affect the activity of evaluated P450. The mean (± standard deviation) values of the % of control activities of CYP1A2 (α-naphthoflavone), CYP2C6 (fluoxetine), CYP2D (quinine) and CYP3A (ketoconazole) model inhibitors at the concentration of 20 µM were: 64.50 (± 1.28), 75.63 (± 3.67), 53.04 (± 4.46), 6.11 (± 1.99) % and 58.16 (± 9.4), 56.12 (± 7.10), 53.63 (± 2.30), 7.95 (± 0.64) % after preincubation.

### Nuclear receptor gene reporter assays

**Fig. 6:**
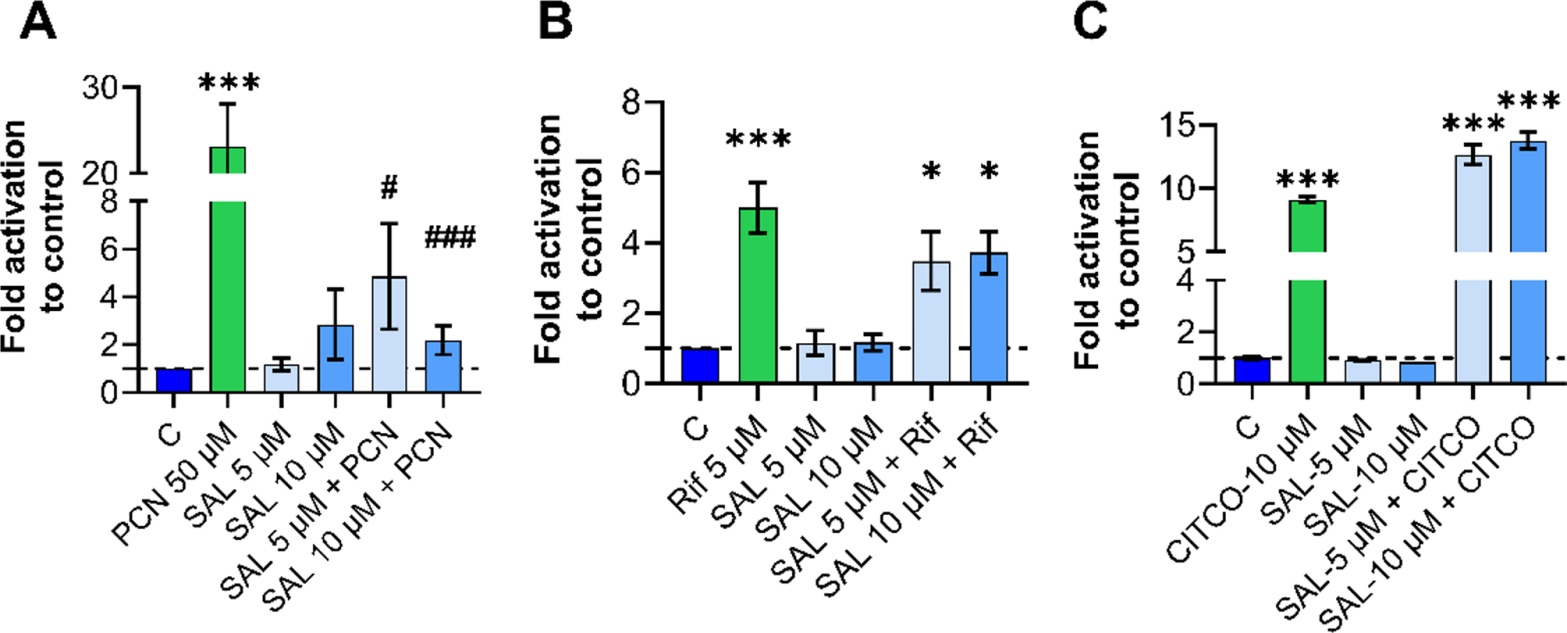
SAL inhibits the activation of rat (**A**) and human (**B**) PXR in luciferase reporter assays conducted in HepG2 cells. Cells were treated with the rat PXR agonist PCN or the human PXR agonist Rif, in combination with SAL (5 and 10 µM). CAR3 activity (**C**) was assessed in a luciferase assay conducted in HepG2 cells. SAL was tested in both agonist and antagonist modes, with CITCO (10 µM) used as a positive control. The agonists significantly induced the activity of the receptors, and in the case of rat PXR, the agonist-induced activity was significantly reduced by both 5 and 10 µM of SAL. All assays were conducted over a 24-hour treatment period. The dashed line represents the maximum effect observed with 0.1% DMSO (C). Statistical significance was determined using ordinary one-way ANOVA followed by Tukey’s multiple comparison test. Results are expressed as the mean ± standard error of the mean of at least three independent experiments. Statistical significance is indicated as follows: * *p* < 0.01, *** *p* < 0.001, compared to the C, # *p* < 0.01, ### *p* < 0.001 compared to agonist (positive control). C – 0.1% DMSO control group, CAR3 – constitutive androstane receptor type 3, CITCO – 6-(4-chlorophenyl)imidazo[2,1-b][1,3]thiazole-5-carbaldehyde O-(3,4-dichlorobenzyl)oxime, DMSO – dimethyl sulfoxide, PCN – pregnenolone-16α-carbonitrile, PXR – pregnane X receptor, Rif – rifampicin, SAL – salidroside

The activation of rat PXR was assessed in the presence of 5 and 10 µM SAL, with or without the agonist PCN at 50 µM. The results indicated that SAL alone did not significantly activate rat PXR (Fig. 6A); however, an unexpected significant inhibition of agonist-induced effect was observed for both concentrations (one-way ANOVA F_5, 12_ = 13.74, *p* = 0.0001). Also, the inhibition of human PXR was evaluated using Rif at 5 µM under similar conditions, as detailed in Fig. 6B. SAL alone did not activate human PXR but exhibited a slight inhibitory effect on Rif-evoked activation of PXR. Furthermore, we investigated the activity of the PXR-related nuclear receptor CAR using the CAR3 variant in a luciferase reporter assay. SAL demonstrated neither agonistic nor antagonistic effects under the assay conditions, while CITCO served as a positive control (Fig. 6C).

### Molecular docking study

A molecular docking study followed by molecular mechanics re-scoring was performed to complement our in vivo and in vitro results and provide insight into the SAL binding with different P450 targets. We docked SAL and P450 respective inhibitors and compared their binding scores. We observed that SAL had worse binding scores than the inhibitors for all targets except CYP3A4/5, indicating weaker inhibitory effects (Table 4). However, for CYP3A4/5, a comparative inhibitor score was unavailable due to the smaller binding cavity of the selected structure (1TQN), as discussed in the ‘Protein Selection and Preparations’ section. The inhibitor ketoconazole was too large to fit into the cavity of this structure, making a direct comparison of binding scores unfeasible.

**Table 4:** Calculated scores, in kcal/mol, of SAL and inhibitors on each protein target; SAL - salidroside

	Score (kcal/mol)	
	SAL	Inhibitor
CYP1A2	-20.5	-27.1
CYP2C9	-18.2	-30.8
CYP2D6	-13.0	-14.4
CYP3A4/5	-17.2	-

**Fig. 7:**
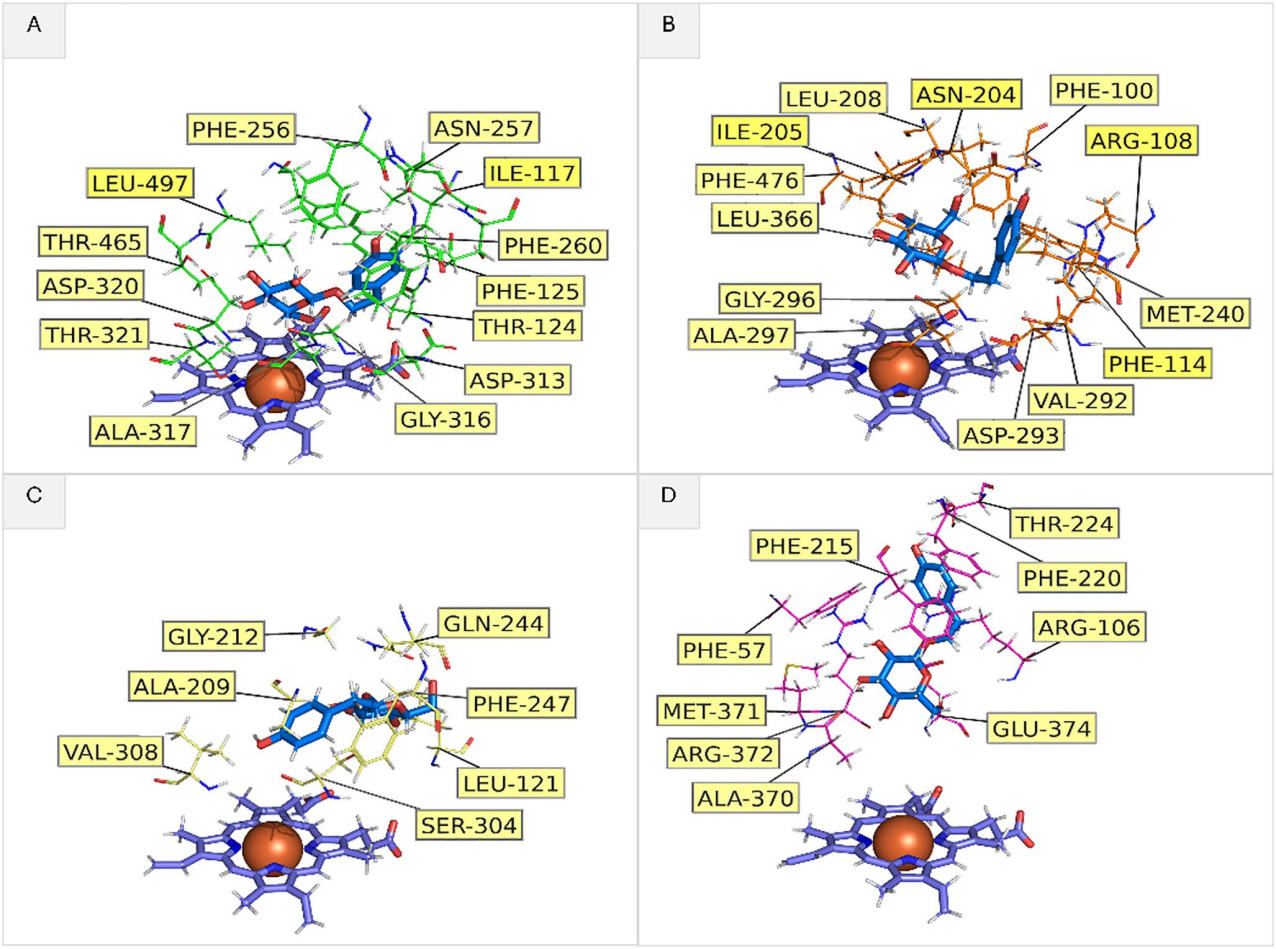
Interactions of SAL with CYP1A2 (**A**), CYP2C9 (**B**), CYP2D6 (**C**), and CYP3A4/5 (**D**). Heme is highlighted as purple sticks with iron atoms as orange spheres, SAL as blue sticks, and interacting residues as lines. SAL – salidroside

**Fig. 8:**
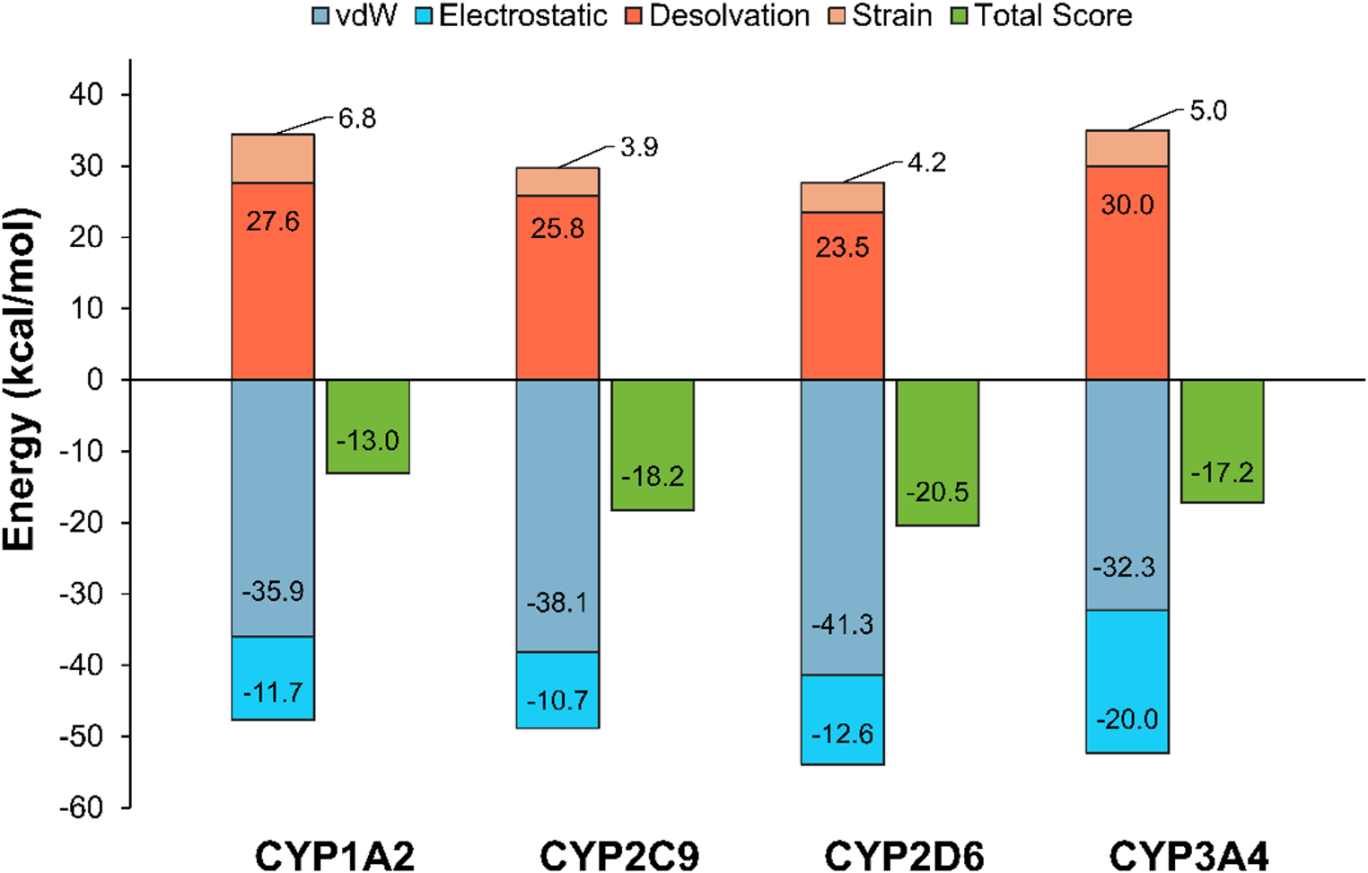
Score decompositions of SAL docked complexes CYP1A2, CYP2C9, CYP2D6, and CYP3A4/5 proteins. SAL – salidroside

SAL interacts primarily with polar amino acid residues located in the access tunnels far from the heme cofactor. This leaves the heme partially accessible from another access tunnel (Fig. 7 B, C, D). The only exception is CYP1A2, where SAL is significantly closer to the heme group because of the space left from the inhibitor furafylline (Fig. 7 A). Nevertheless, the binding score shows very low affinity, indicating poor complementarity (Fig. 8). The energy decomposition of individual complexes shows quite similar results. Although SAL had higher values for attractive terms such as electrostatic and van der Waals interactions, overall scores were significantly reduced due to ligand strain and high desolvation penalties (Fig. 8). This indicates a very low preference for SAL to bind in the tunnel or binding site of all studied P450. The results of the in silico experiment were in accordance with the results of inhibitory assays.

## Discussion

The toxicity and the risk of drug interactions of new drugs are usually tested during the preclinical phase of drug development. Unequivocal safety data are often missing in the case of herbs and herbal preparations. These traditional medicines rely primarily on empirical knowledge and do not undergo a standard preclinical development phase. Similarly, food supplements based on traditional medicine containing herbal active principles are not tested for drug interactions as medicinal products. SAL is a glycoside and one of the main active principles of the herb *R. rosea*, which is used in traditional medicine and is a constituent of various food supplements. According to our results, SAL seems to interact neither with rat (both in vivo and in vitro) nor with human P450 (in vitro) in a substantial way, and might be considered safe regarding the combinations with drugs that are P450 substrates. SAL also prevented agonist-mediated PXR induction. Moreover, additional biochemical and physiological evaluations found no signs of SAL toxicity in the animal model when administered orally for seven consecutive days at a dose of up to 45 mg/kg/day.

The content of SAL in *R. rosea* roots and rhizomes depends on environmental conditions and ranges from 0.05 to 2.88% of dry plant material [59–61]. Similar concentrations can also be found in *R. rosea* extracts [62]. Recommended daily dose of the registered herbal medicinal product containing WS 1375, a patented *R. Rosea* extract (3.0–8% of rosavins and > 1% of SAL [63]), is 400 mg [64] representing daily dose of SAL around 4 mg that is around 0.6 mg/kg. The doses of SAL used in our work (5–45 mg/kg) are well above the probable intake from dietary supplements or diet in humans, and align with the study’s aim of assessing P450-mediated herb-drug interaction potential. Moreover, the selected dose range also corresponds to that used in animal studies testing its antidepressant-like effects [9, 28].

In humans, the maximal plasma level (948 ng/mL) is reached 2 h after oral administration of SAL at a dose of 9.34 mg [22]. The ICH M12 guideline on drug interaction studies recommends a high concentration of the tested substance (e.g., 50 times the maximal plasma level) for in vitro inhibition studies [65], which is approximately 150 µM on the results of Panosssian et al. [22]. Our in vitro inhibition studies covered the ranges from 1.0 to 100.0 µM for RLMs and 0.5 to 500.0 µM for HLMs, respectively.

The influence of drugs on P450 activity can be caused by mechanisms other than direct binding of the drug to the substrate pocket or the allosteric site. Some herbal constituents like hyperforin from St. John’s wort can influence the P450 activity via interaction with nuclear receptors regulating the gene expression of these enzymes. Other substances can affect the levels of endogenous ligands of these receptors (hormones, cytokines) or act via different mechanisms (enzyme stabilisation, increased translational efficiency, etc.). Because of that, it is necessary to evaluate the impact of the tested substance in SAL-treated animals in addition to the in vitro testing.

Seven-day administration of SAL to rats at doses of 5, 15 and 45 mg/kg of body weight/day did not show any toxicity effects. A complete blood count and selected biochemical parameters studied in our experiment were within the physiologic range for male Wistar rats [66–68]. Also, the total body weight and daily weight gain agree with the standard growth curves [69]. A study on the safety of SAL in rats reports similar weight gains and total body weights, even though SAL was administered at doses from 500 to 2000 mg/kg/day for 28 days. The no-observed-adverse-effect level (NOAEL) for SAL was assessed at least 2000 mg/kg/day [67]. Olsson et al. describe no severe side effects of ethanolic and water extracts in humans administered at the dose of 567 mg/day for 28 days or at the doses of 340 or 680 mg per day over six weeks [70]. The only adverse effects reported after human use of *R. rosea* extract were headache, nausea and hypersensitivity, which is mentioned in the Summary of Product Characteristics (SmPC) of the registered traditional herbal medicine with *R. rosea* [64]. Although comprehensive safety data, including that pertaining to special populations, is limited, the overall conclusion of European Union Herbal monograph on *R. rosea L. rhizome et radix* is that using products containing *R. rosea* is not harmful when used in specified conditions [7].

Our findings confirm the hypothesis that protein and P450 content differences among groups could cause discrepancies between molecular and specific metabolic activities. Although the calculation of molecular activities nullified the statistical significance of the differences between groups, slightly elevated activities in SAL groups indicated possible changes in gene expression or particular enzyme amounts. Therefore, these parameters were evaluated in CYP1A2 and CYP2C, where specific activities in group SAL5 were significantly changed. The mechanism of slightly increased metabolic activity in group SAL5 can be caused either by the allosteric activation of P450 or by an increase in particular P450 amounts. The allosteric interaction requires the binding of SAL to the P450 structure. During RLMs preparation, microsomes are washed several times with various buffers, and allosteric modulators should be removed unless they bind to P450 irreversibly. Allosteric activation should also be present in in vitro inhibitory studies when SAL is added with P450 into the reaction mixture. Nevertheless, this was not found in neither HLMs nor RLMs in vitro studies. Thus, the allosteric activation mechanism can be excluded.

The results of the molecular docking study supported our findings. Although this rigid protein docking approach employed here does not account for P450 conformational flexibility, the qualitative agreement between the in silico predictions and experimental data, which showed no direct inhibition, reinforces the conclusion that SAL has low affinity for P450 binding sites.

There are also other possible mechanisms to increase P450 concentrations. Similarly to allosteric activation, the substance can bind to the structure of P450 without impacting the catalytic activity but stabilising the enzyme and preventing its biodegradation. Another possibility requires binding of the substance to nuclear receptors that regulate gene expression of particular P450. Protein and mRNA amounts could be compared to distinguish between these two mechanisms. The change in the gene expression could be interpreted as possible activation or blockade of nuclear receptors. In contrast, the isolated increase in the protein amount without change in the mRNA levels could indicate protein stabilisation. PXR is a nuclear receptor involved in drug-herb interactions via the regulation of CYP3A and PgP expression. Interestingly, even though SAL did not exhibit inhibitory effect itself, it diminished the effect of both rat and human PXR inducers. Therefore, it showed protective effect against PXR-mediated CYP3A4 induction, preserving the constitutive CYP3A metabolic activity. Similar effect was also observed for other compounds of natural origin such as resveratrol [71], silybin, isosilybin [72], or camptothecin [73]. The possible mechanisms might be competitive or allosteric antagonism, interference with coactivators or the effect on posttranslational phosphorylation-mediated mechanisms and signalling pathways [74, 75]. SAL showed no agonist or antagonist activity against human CAR3 receptor. Moreover, neither the change in protein content nor mRNA levels of investigated isoforms were detected, which is in line with the gene reporter assays.

The metabolic activity is based exclusively on the conversion of the substrate by P450 molecules. In most cases, SAL increased metabolic activities between 5 and 30% over the activity of controls. The clinical implications of such an increase would be low and within the range of usual interindividual variability in the drug metabolism that concerning individual enzymes can reach from 30- to 100-fold variation depending on a single P450 [76]. But since sex-specific differences in P450 are described, it would be valuable to perform the in vivo experiment with female rats as well [77].

The data on the influence of SAL on P450 from in vivo models are scarce. Panossian et al. tested the extracts from *R. rosea* with 2.5–2.7% of SAL in male rats. The extracts were administered by oral gavage at 50 mg/kg/day for three days. The pharmacokinetics of theophylline and warfarin, the substrates of CYP2C11 + CYP1A2 and CYP2C11 + CYP3A, respectively, were evaluated [78]. The extracts did not affect the pharmacokinetic parameters of both drugs being assessed, which aligns with our results. The daily dose of SAL used in this experiment (1.25–1.35 mg/kg) was much lower than in our work. Also, the in vivo model of pharmacokinetics used by Panossian et al. includes more factors that can influence drug pharmacokinetics, except for P450 enzymes. Another work used female rabbits to study the influence of *R. rosea* extract on the metabolism of losartan by P450. The extract was administered in a single dose of 50 mg/kg orally 15 min before losartan, and the plasma levels of the drug and its active metabolite EXP3174 (CYP2C9 and CYP3A4) were analysed. The authors conclude that a potential interaction of losartan with *R. rosea* may occur. The extract caused an almost 2-fold increase in the area under the curve and decreased apparent total body clearance of losartan [79]. The role of P450 in this effect is disputable as none of the pharmacokinetic parameters of the metabolite was significantly changed. Finally, Wei et al. tested SAL in male Sprague-Dawley rats. The 30 mg/kg dose was administered either for seven days or as a single dose by oral gavage. The metabolic activity of CYP1A2, CYP2B6, CYP2C, CYP2D, and CYP3A were assessed using the cocktail approach. The induction of CYP1A2, CYP2B6, CYP2C, and CYP3A was found [80], which would be in line with our specific activity results. Nevertheless, the methodology of this study raises some concerns. The animal experiment design describes administering a single dose of SAL to controls, 30 min before administering the cocktail probe.

The rat is a conventional model for P450 interaction studies. The sequential homology between rat and human P450 isoforms is high but not absolute: 70% for CYP1A2, 75% for CYP2C6, 71% for CYP2D1/2, 73% for CYP3A1/2 [81]. Nevertheless, the substrate specificity may vary (e.g. lidocaine is metabolised by CYP2C11 in rats and by CYP3A4 in humans) [82]. In addition, the metabolic activity is influenced by different inducers and inhibitors, as described in detail by Martignoni et al. [83]. For these reasons, it was necessary to perform inhibition studies in both RLMs and HLMs. The results of in vitro experiments with HLMs and RLMs indicate that neither inhibition nor allosteric activation of P450 by SAL is probable. Other authors report inhibitory effects of either *R. rosea* extracts or extracts prepared from commercially available products containing this herb on human recombinant CYP1A2, CYP2C9, CYP2D6 and CYP3A4 [84–87]. Some of these experiments also analysed the effect of major active constituents of *R. rosea*, including SAL. They concluded that SAL, rosavin, rosarin, rosin, and tyrosol are not responsible for inhibiting P450 [84, 87]. Our results are complementary to the research of Kasprzyk et al. (2023). In their study, no inhibitory effects of SAL were detected on CYP1A2, CYP2B6, CYP2C8, CYP2C9, CYP2C19, CYP2D6, or CYP3A4/5 up to a concentration of 50 µM in HLMs. Moreover, they observed no induction of CYP1A2, CYP2B6, or CYP3A4 in human hepatocytes [88]. Consistent with these observations, our data show minimal induction and reveal no inhibitory effect in HLMs even at a SAL concentration of 500 µM.

Based on our data, SAL or *R. Rosea* extract might be considered safe regarding the combinations with drugs that are P450 substrates.

## Supplementary Information

Below is the link to the electronic supplementary material.

Supplementary Material 1

## Data Availability

The datasets generated and/or analysed during the current study are available in the [Mendeley data] repository [https://data.mendeley.com/datasets/p8hw5rt8k4/2 ] [58].

## Abbreviations

ALP: Alkaline phosphatase
ALT: Alanine transaminase
AST: Aspartate transaminase
β-actin: Beta actin
C: Control group
CAR: Constitutive androstane receptor
CITCO: 6-(4-chlorophenyl)imidazo[2,1-b][1,3]thiazole-5-carbaldehyde O-(3,4-dichlorobenzyl)oxime
CZE: Czech Republic
DMSO: Dimethyl sulfoxide
FIN: Republic of Finland
FRA: French Republic
GAPDH: Glyceraldehyde-3-phosphate dehydrogenase
GER: Federal Republic of Germany
GGT: Gamma-glutamyl transferase
HLMs: Human liver microsomes
JPN: Japan
MCH: Mean corpuscular haemoglobin
MCHC: Mean corpuscular haemoglobin concentration
MCV: Mean corpuscular volume
MPV: Mean platelet volume
NOAEL: No observed adverse effect level
P450: Cytochromes P450
PCN: Pregnenolone-16α-carbonitrile
PCT: Plateletcrit
PDW: Platelet distribution width
PL: Republic of Poland
PLANTS: Protein-Ligand ANT System
PRC: People’s Republic of China
PXR: Pregnane X receptor
qRT-PCR: Real-time quantitative reverse transcription polymerase chain reaction
RDW: Red blood cell distribution width
Rif: Rifampicin
RIN: RNA integrity number
RLMs: Rat liver microsomes
SAL: Salidroside
SAL5: Animal group administered with salidroside 5 mg/kg of body weight/day
SAL15: Animal group administered with salidroside 15 mg/kg of body weight/day
SAL45: Animal group administered with salidroside 45 mg/kg of body weight/day
SGLT1: Sodium-dependent glucose cotransporter 1
SmPC: Summary of product characteristics
USA: United States of America
