# Supplementary material for: The effect of salidroside, an active component of Rhodiola rosea, on the metabolic activity of rat and human cytochromes P450 in preclinical studies
Source: Pharmacol Rep. 2026 Mar 11;78(3):808–25. doi: 10.1007/s43440-026-00842-w (PMC13275619; doi:10.1007/s43440-026-00842-w)
Supplement: Supplementary file 1 — Supplementary Material 1 [file 43440_2026_842_MOESM1_ESM.pptx]

## Slide 1
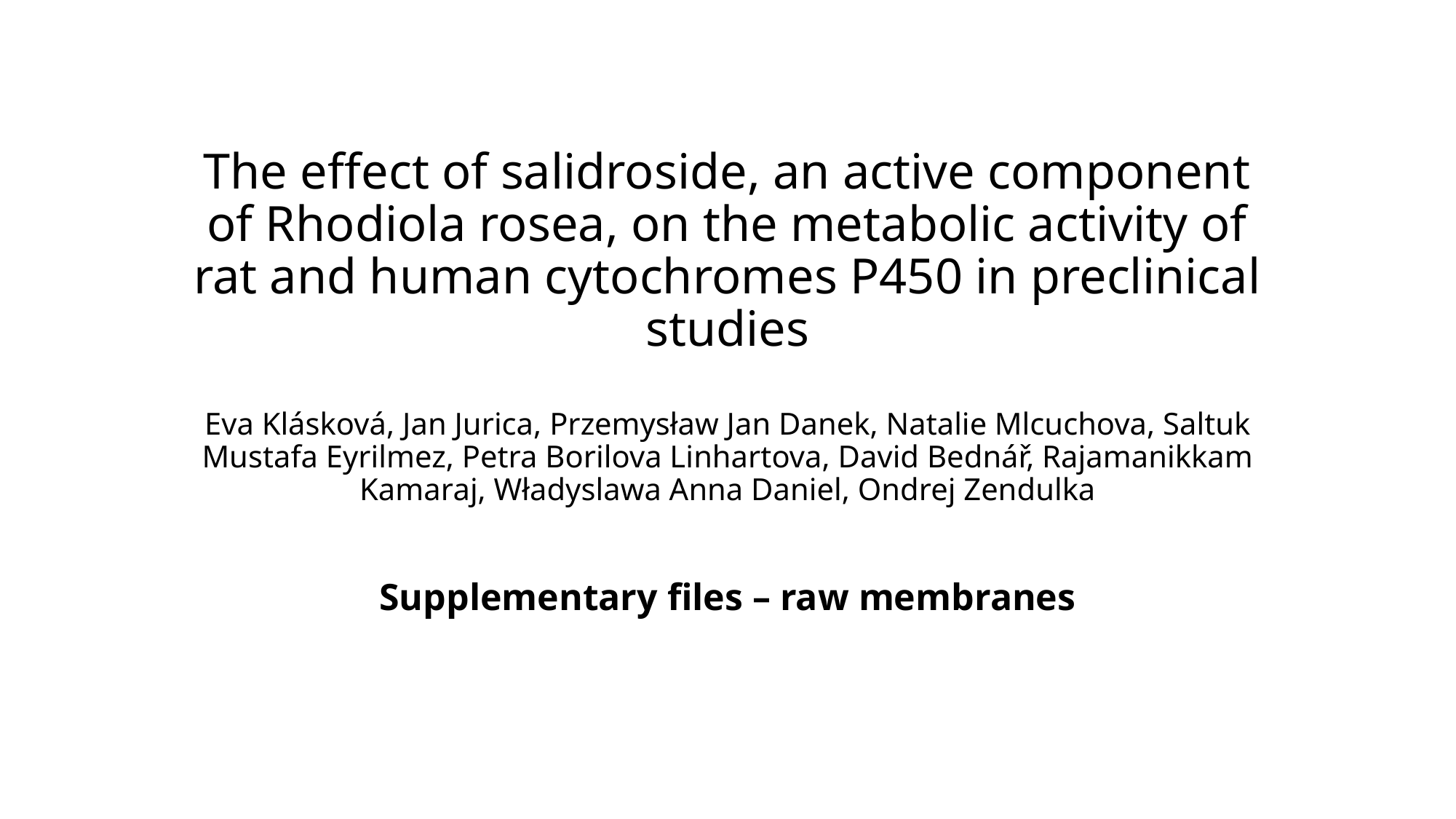

# The effect of salidroside, an active component of Rhodiola rosea, on the metabolic activity of rat and human cytochromes P450 in preclinical studies Eva Klásková, Jan Jurica, Przemysław Jan Danek, Natalie Mlcuchova, Saltuk Mustafa Eyrilmez, Petra Borilova Linhartova, David Bednář, Rajamanikkam Kamaraj, Władyslawa Anna Daniel, Ondrej Zendulka
Supplementary files – raw membranes

## Slide 2
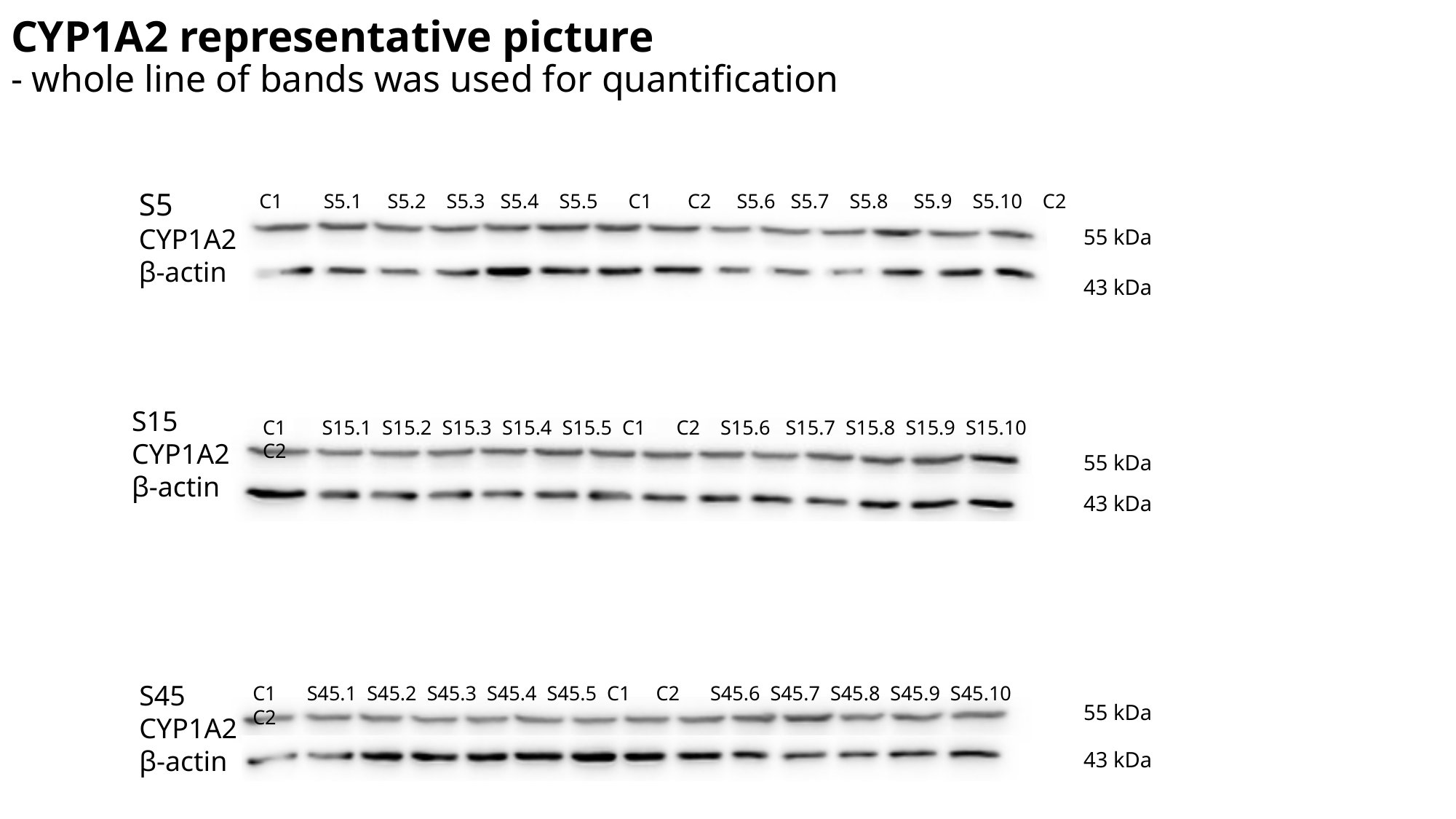

# CYP1A2 representative picture - whole line of bands was used for quantification
S5
CYP1A2
β-actin
C1 S5.1 S5.2 S5.3 S5.4 S5.5 C1 C2 S5.6 S5.7 S5.8 S5.9 S5.10 C2
55 kDa
43 kDa
S15
CYP1A2
β-actin
 C1 S15.1 S15.2 S15.3 S15.4 S15.5 C1 C2 S15.6 S15.7 S15.8 S15.9 S15.10 C2
55 kDa
43 kDa
S45
CYP1A2
β-actin
C1 S45.1 S45.2 S45.3 S45.4 S45.5 C1 C2 S45.6 S45.7 S45.8 S45.9 S45.10 C2
55 kDa
43 kDa

## Slide 3
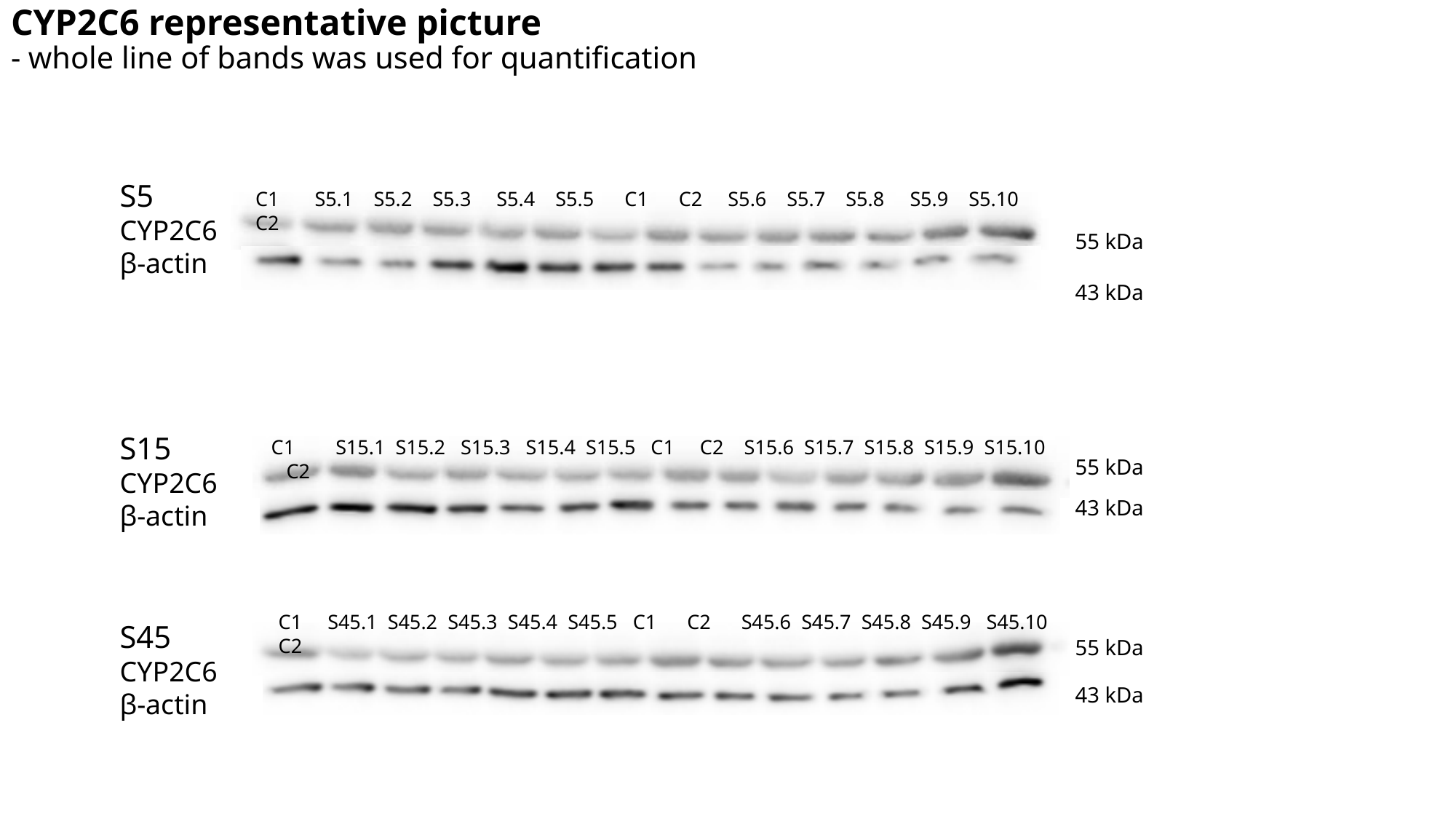

# CYP2C6 representative picture - whole line of bands was used for quantification
S5
CYP2C6
β-actin
C1 S5.1 S5.2 S5.3 S5.4 S5.5 C1 C2 S5.6 S5.7 S5.8 S5.9 S5.10 C2
55 kDa
43 kDa
S15
CYP2C6
β-actin
C1 S15.1 S15.2 S15.3 S15.4 S15.5 C1 C2 S15.6 S15.7 S15.8 S15.9 S15.10 C2
55 kDa
43 kDa
C1 S45.1 S45.2 S45.3 S45.4 S45.5 C1 C2 S45.6 S45.7 S45.8 S45.9 S45.10 C2
S45
CYP2C6
β-actin
55 kDa
43 kDa

## Slide 4
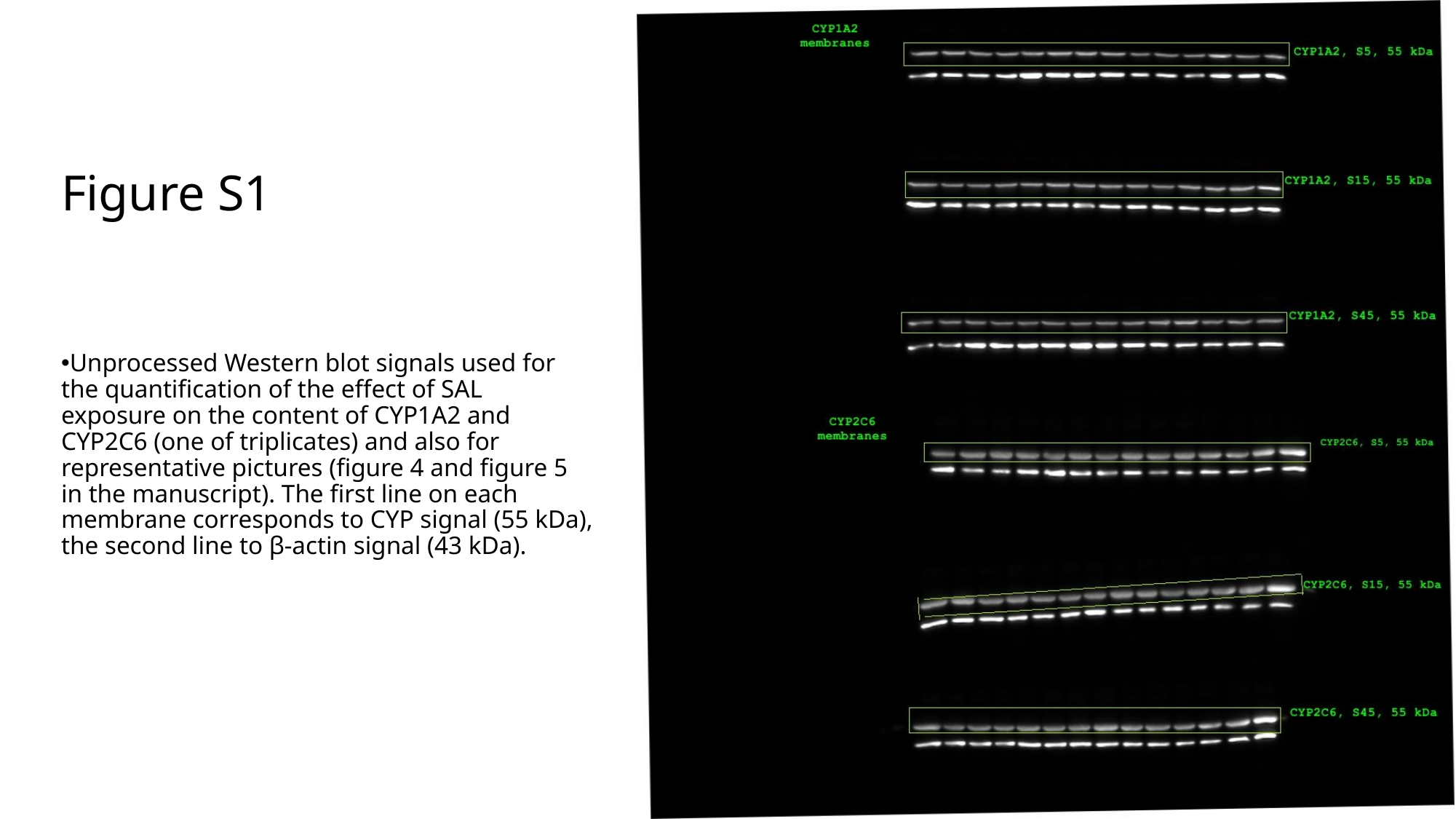

# Figure S1
Unprocessed Western blot signals used for the quantification of the effect of SAL exposure on the content of CYP1A2 and CYP2C6 (one of triplicates) and also for representative pictures (figure 4 and figure 5 in the manuscript). The first line on each membrane corresponds to CYP signal (55 kDa), the second line to β-actin signal (43 kDa).

## Slide 5
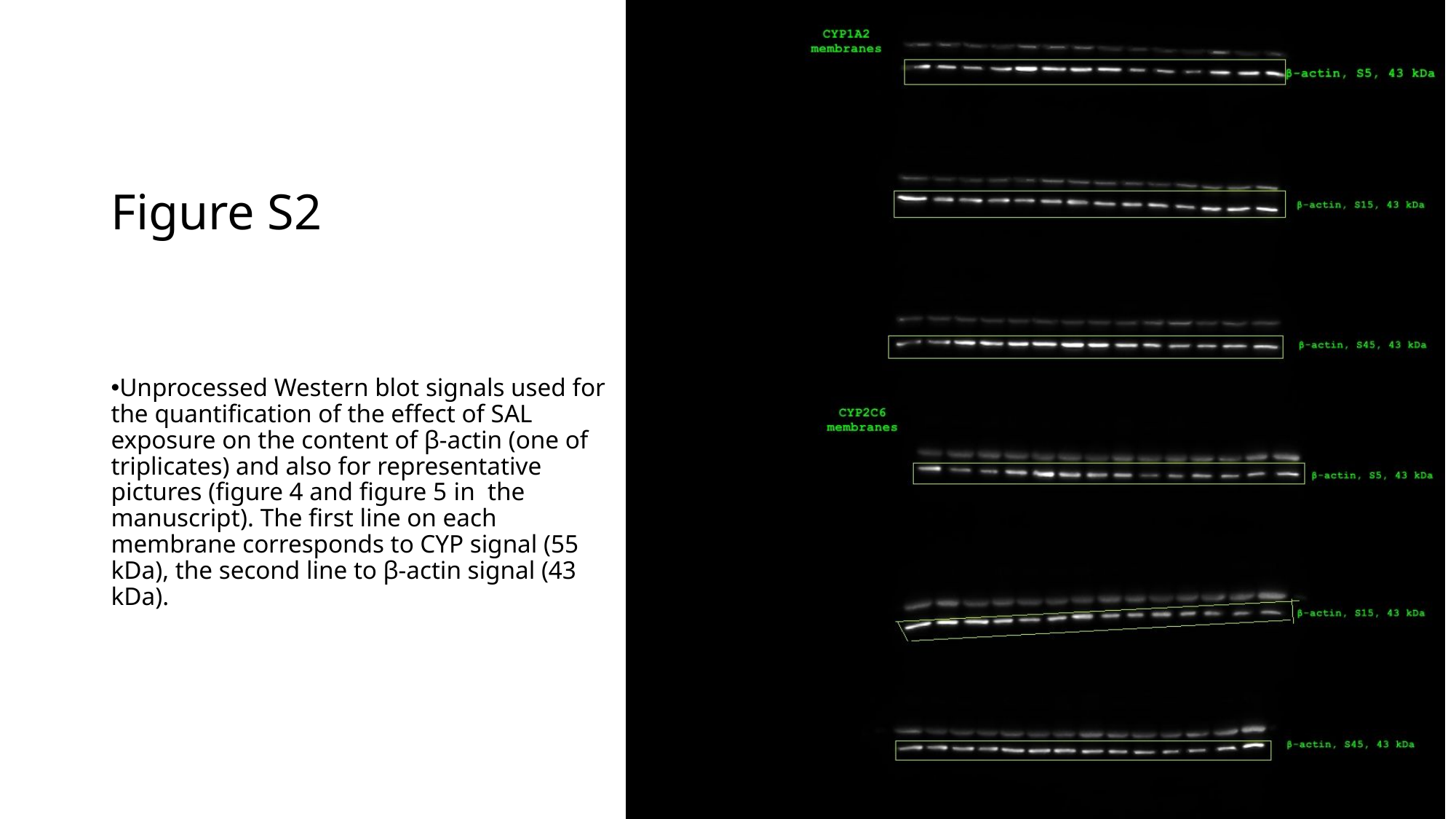

# Figure S2
Unprocessed Western blot signals used for the quantification of the effect of SAL exposure on the content of β-actin (one of triplicates) and also for representative pictures (figure 4 and figure 5 in the manuscript). The first line on each membrane corresponds to CYP signal (55 kDa), the second line to β-actin signal (43 kDa).

## Slide 6
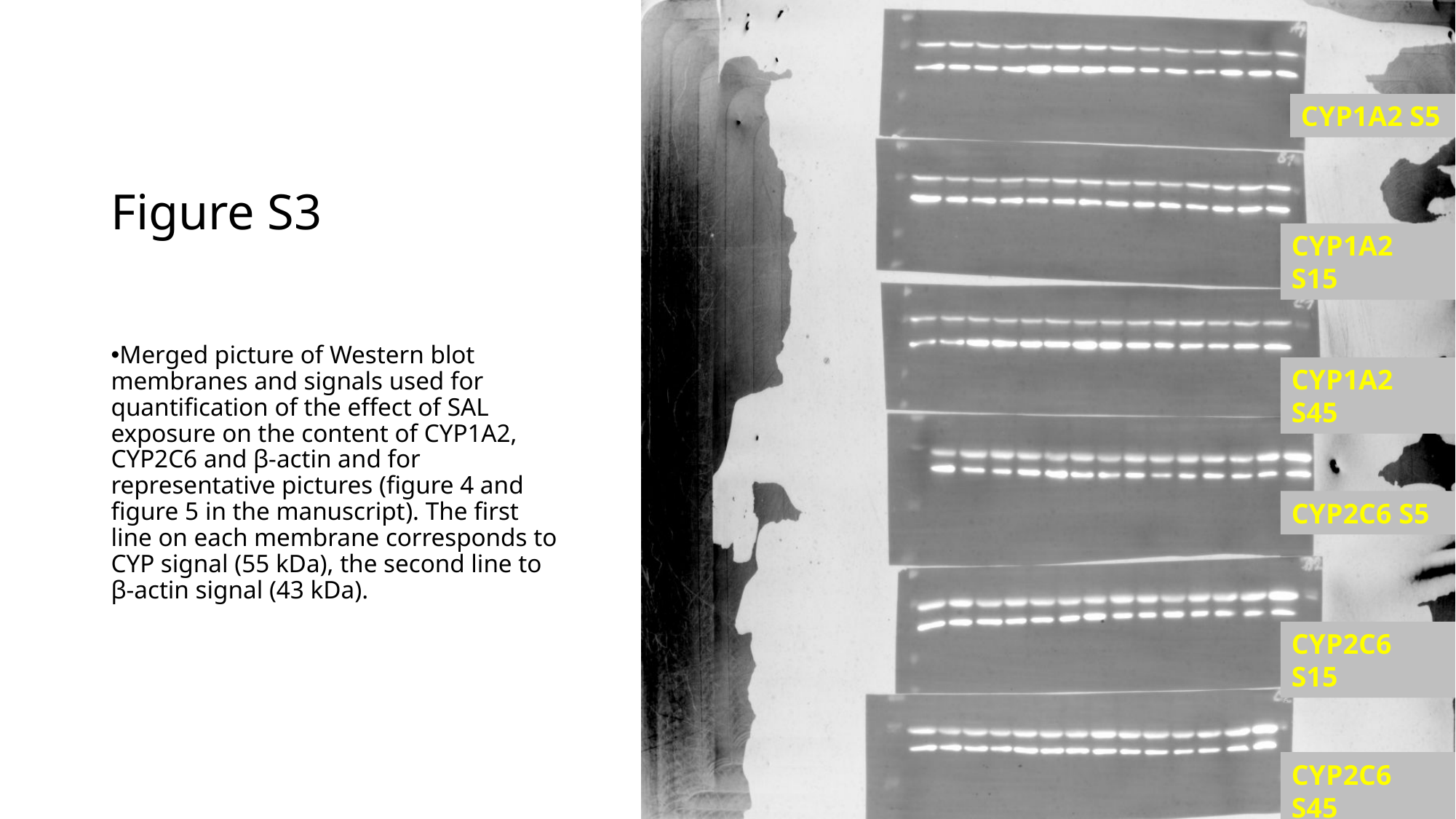

# Figure S3
CYP1A2 S5
CYP1A2 S15
Merged picture of Western blot membranes and signals used for quantification of the effect of SAL exposure on the content of CYP1A2, CYP2C6 and β-actin and for representative pictures (figure 4 and figure 5 in the manuscript). The first line on each membrane corresponds to CYP signal (55 kDa), the second line to β-actin signal (43 kDa).
CYP1A2 S45
CYP2C6 S5
CYP2C6 S15
CYP2C6 S45

## Slide 7
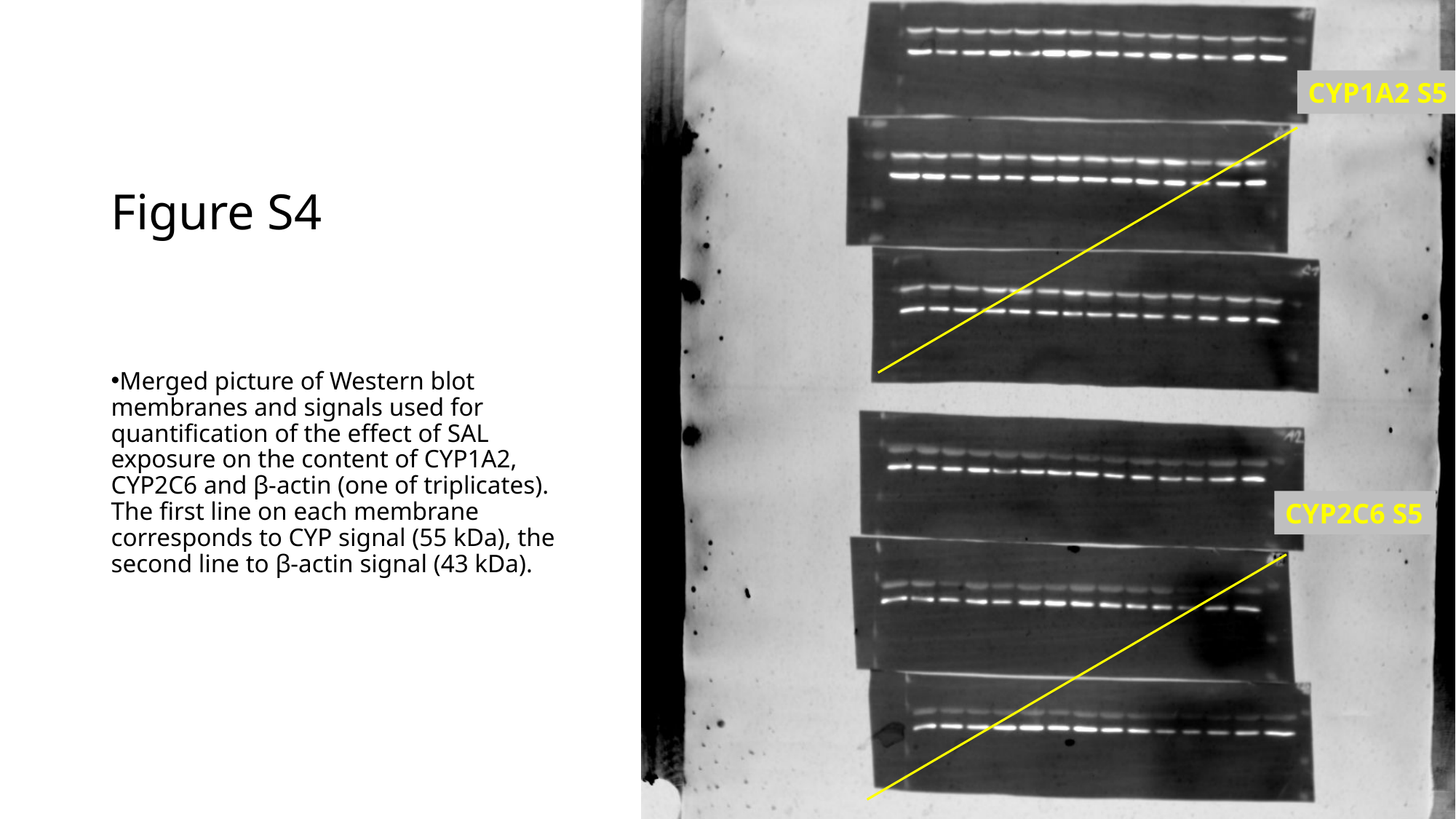

# Figure S4
CYP1A2 S5
Merged picture of Western blot membranes and signals used for quantification of the effect of SAL exposure on the content of CYP1A2, CYP2C6 and β-actin (one of triplicates). The first line on each membrane corresponds to CYP signal (55 kDa), the second line to β-actin signal (43 kDa).
CYP2C6 S5

## Slide 8
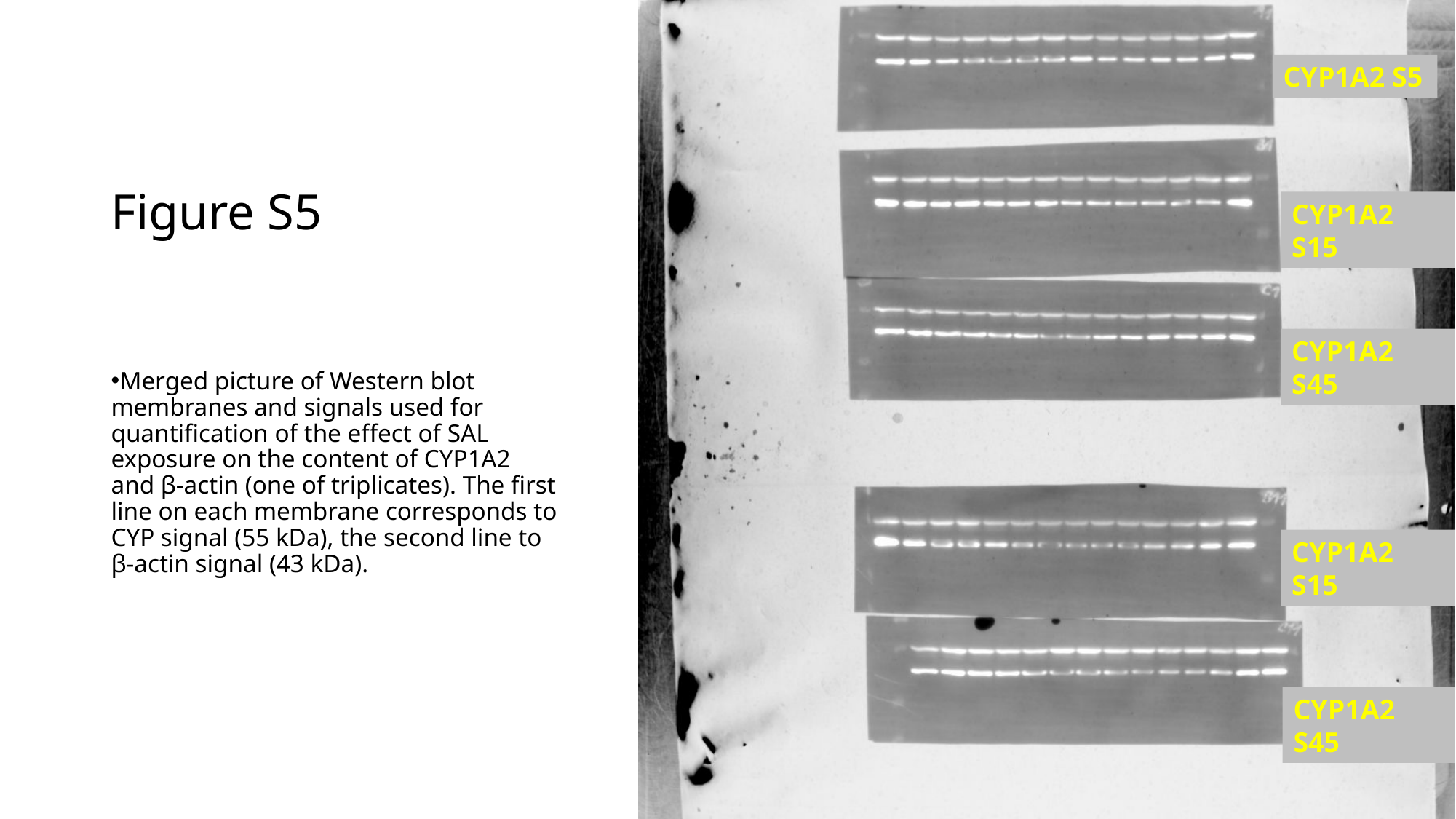

# Figure S5
CYP1A2 S5
CYP1A2 S15
Merged picture of Western blot membranes and signals used for quantification of the effect of SAL exposure on the content of CYP1A2 and β-actin (one of triplicates). The first line on each membrane corresponds to CYP signal (55 kDa), the second line to β-actin signal (43 kDa).
CYP1A2 S45
CYP1A2 S15
CYP1A2 S45

## Slide 9
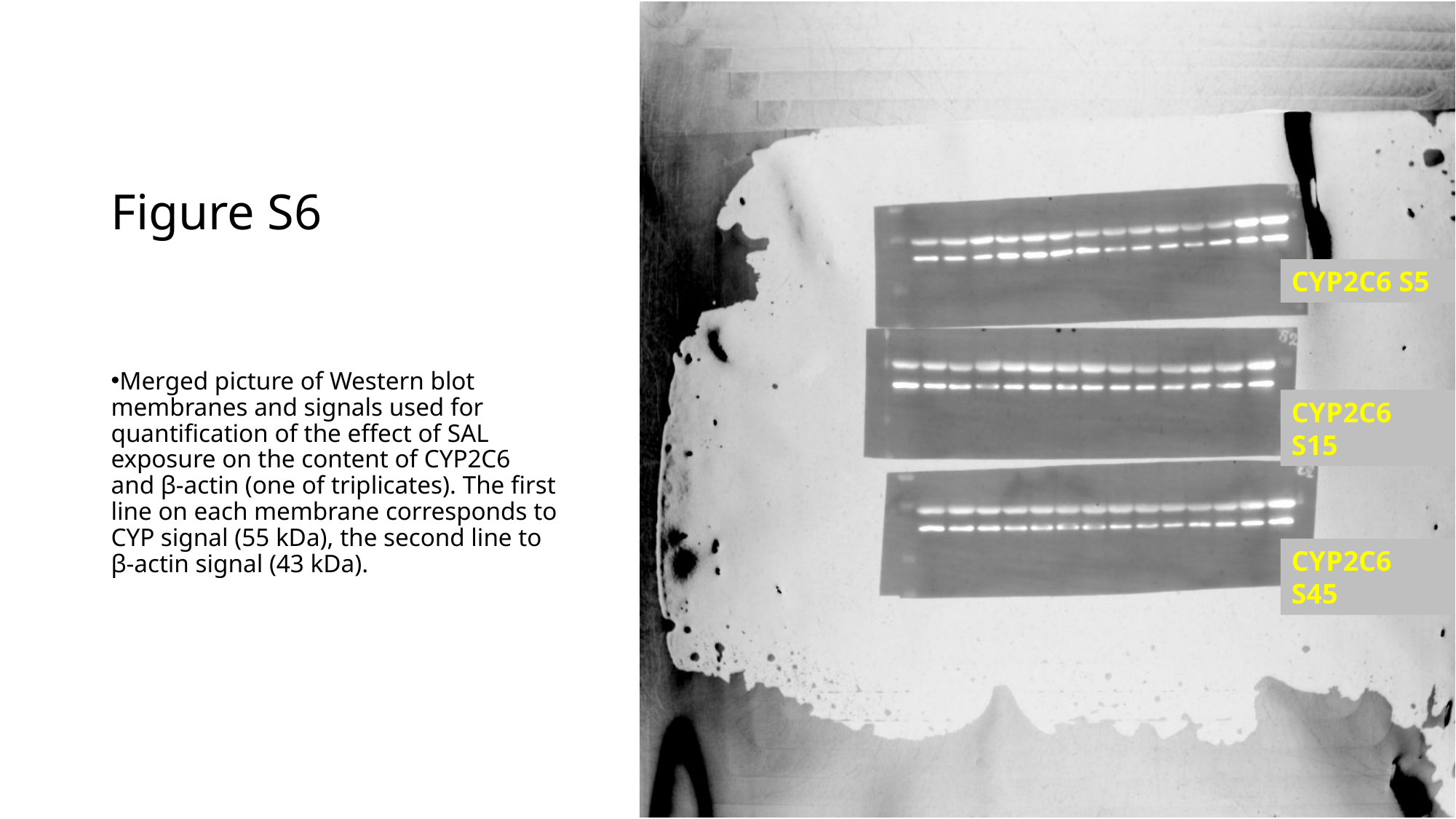

# Figure S6
Merged picture of Western blot membranes and signals used for quantification of the effect of SAL exposure on the content of CYP2C6 and β-actin (one of triplicates). The first line on each membrane corresponds to CYP signal (55 kDa), the second line to β-actin signal (43 kDa).
CYP2C6 S5
CYP2C6 S15
CYP2C6 S45

## Slide 10
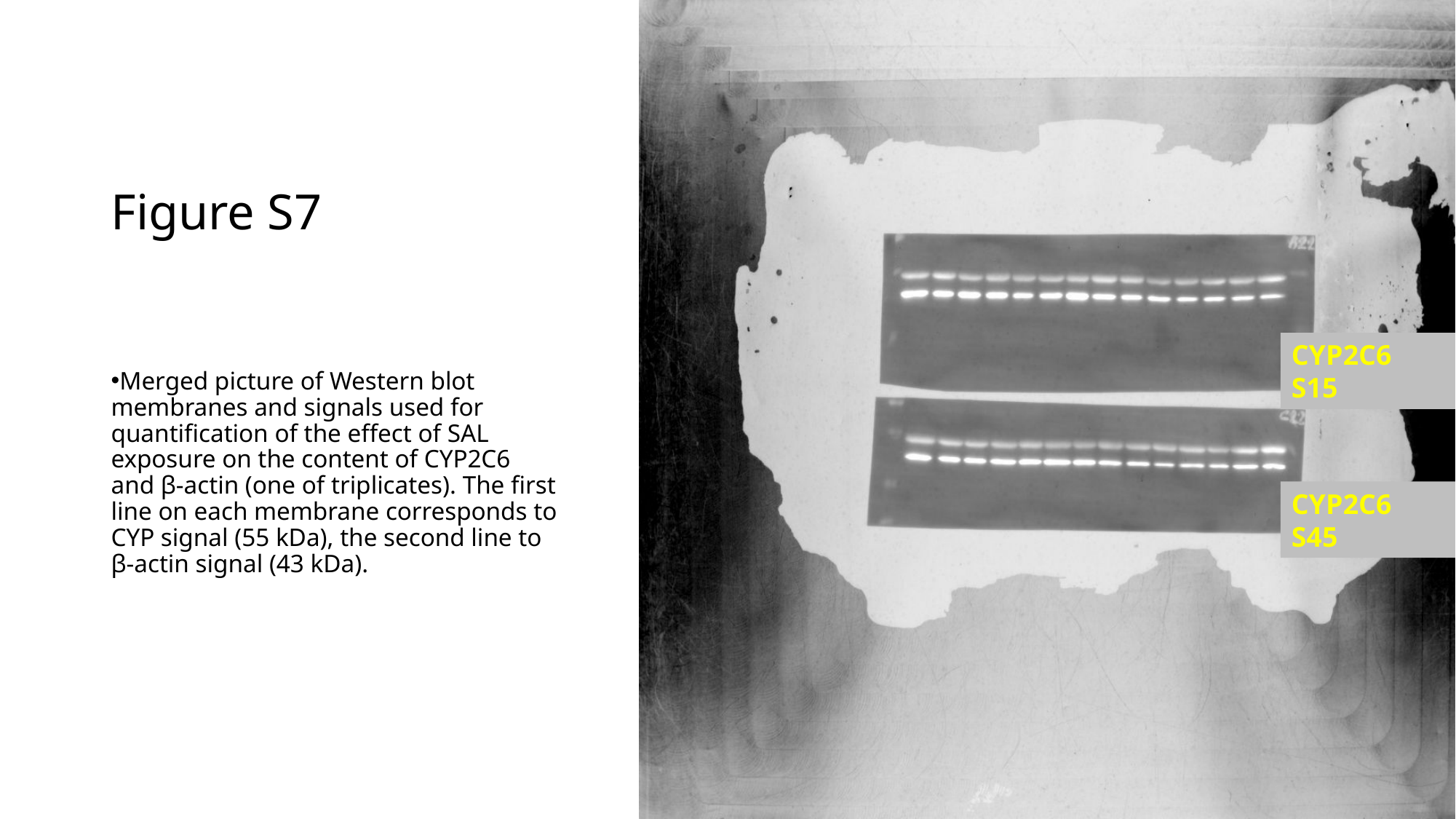

# Figure S7
Merged picture of Western blot membranes and signals used for quantification of the effect of SAL exposure on the content of CYP2C6 and β-actin (one of triplicates). The first line on each membrane corresponds to CYP signal (55 kDa), the second line to β-actin signal (43 kDa).
CYP2C6 S15
CYP2C6 S45
